# A thermodynamically favoured molecular computer

**DOI:** 10.1038/s41586-026-10996-5

**Published:** 2026-09-16

**Authors:** Tristan Stérin, Abeer Eshra, Constantine Glen Evans, Janet Adio, Damien Woods

**Affiliations:** 1 Hamilton Institute and Department of Computer Science, Maynooth University, Kildare, Ireland; 2 prgm.dev, Paris, France; 3 Evans Foundation for Molecular Medicine, Pasadena, CA USA; 4 Department of Biology, Maynooth University, Kildare, Ireland

**Keywords:** DNA computing, Computer science

## Abstract

Computers, like life, are usually out of equilibrium^1,2^. Undesired error states are thwarted by energetically costly kinetic control processes: proofreading of biological polymers, error correction in computing and redundancy in molecular programming. Unlike life as we know it, theory shows that computation can be embedded in a system relaxing to a thermodynamically favoured equilibrium state^3,4^. Machine learning and search algorithms use this idea^5,6^, although executed on non-equilibrium architectures at enormous energy cost. Physically implementing thermodynamically favoured computation requires a programmable medium amenable to energy landscape engineering. Here we demonstrate a thermodynamically favoured Scaffolded DNA Computer (SDC) on 10 programs, including Multiplication-by-3, Division-by-2, 8-bit Parity-detection and Addition of 25-bit numbers—a 100-bit computation. SDC algorithms have simple experimental protocols, can be reused dozens of times and small instances run in under a minute. Mathematical, physical and computer science principles explain why the SDC is thermodynamically favoured, why it does not require error-correction or precise kinetic control, and how it is programmable and scalable. This work creates a new way to think about equilibrium computation in all manner of synthetic systems.

## Abstract (web summary)

Scaffolded DNA Computer performs diverse computations while thermodynamically relaxing towards equilibrium, demonstrating simplicity, reusability, speed, robustness and scalability of the approach by running 10 programs, including parity, multiplication and 25-bit addition.

## Main

**Fig. 1:**
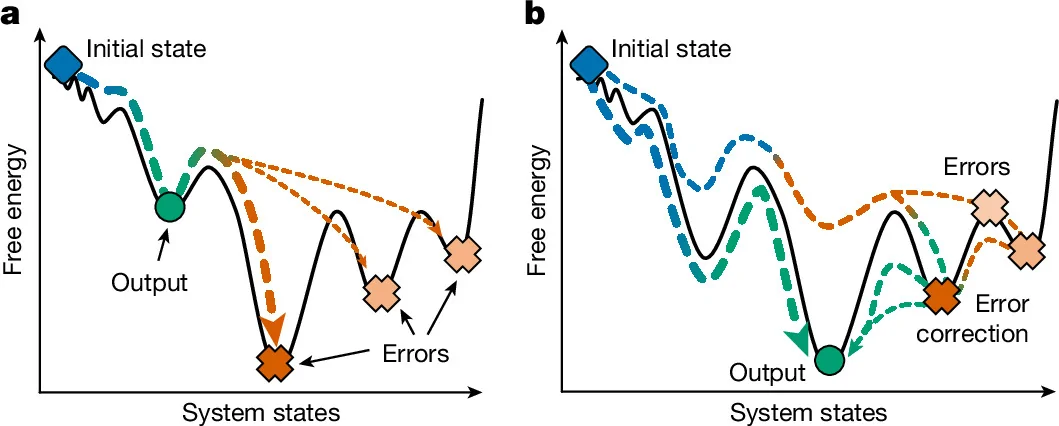
Comparison of classical and thermodynamically favoured computation. **a**, Classical molecular computers have an intended target or final state that is out of equilibrium. Digital electronic computers have similar issues, in which decades of research have lowered error rates but with large energetic costs^7^. **b**, Thermodynamically favoured computation has the output state being the energetically most favoured. Also, input states should be easy to prepare, and the landscape should be efficiently navigable. Hence, computation happens by the system naturally and automatically going to equilibrium (Supplementary Notes 1 and 2).

Analogous to living systems that consume fuel to stave off thermodynamic heat death, computers are typically out of equilibrium, consuming energy to enforce logical correctness. The massive gap between modern computing energy consumption^7,8^ and minimal theoretical requirements^2,4,9,10^ leaves substantial room to rethink computer design. A perhaps counter-intuitive avenue is to design a programmable physical system whose equilibrium encodes the output of an algorithm^3^, allowing the computer to simply drift to the right answer (Fig. 1 and Supplementary Note 1). This notion has appeared in various forms^3,4,7,11^ but contrasts with popular computing methods; however, it is still not widely adopted. There are several challenges: we need a physical implementation that is computationally expressive and programmable, and has easily prepared initial states, and a controllable energy landscape for rapid navigation to target outputs with high probability.

Using DNA^12^, small teams of molecular programmers demonstrated self-assembling 6-bit programs^13^, pattern recognizers^14^, self-replicators^15^, timers in cell culture^16^, analog dynamical systems^17^, Boolean circuits^18–22^ and robots^23,24^. Out-of-equilibrium design principles underlie these impressive achievements, but create challenges and off-target interactions, including unintended nucleation in algorithmic self-assembly, leaks or errors in strand displacement systems, tedious manual preparation and sensitivity to experimental conditions. However, several inspirational studies show that computation can be encoded in chemical equilibria, theoretically and experimentally^3,25,26^. Although DNA origami^27^ lacks any notion of computation, its beautiful scaffolded principle^27^ provides inspiration through a huge thermodynamic force that drives nanostructure assembly^28^ (Supplementary Note 2).

To address the stated challenges of thermodynamically favoured computation, we propose and demonstrate the Scaffolded DNA Computer (SDC) model of computation. Our approach is principled: mathematically, in simulation and by molecular design, correct computation outputs are highly favoured, outcompeting exponentially many off-target configurations. The SDC has an efficiently traversable energy landscape yielding highly parallel kinetics with multiple potential pathways to the output. The design has thermodynamic costs but, unlike typical forms of computation, avoids the need for explicit error-correction subsystems by naturally evolving to the desired output state at equilibrium (Fig. 1b). By rethinking computation to be thermodynamically favoured, we get a conceptually simple system that is programmable, robust to errors, fast, reusable and scalable.

## SDC design principles

**Fig. 2:**
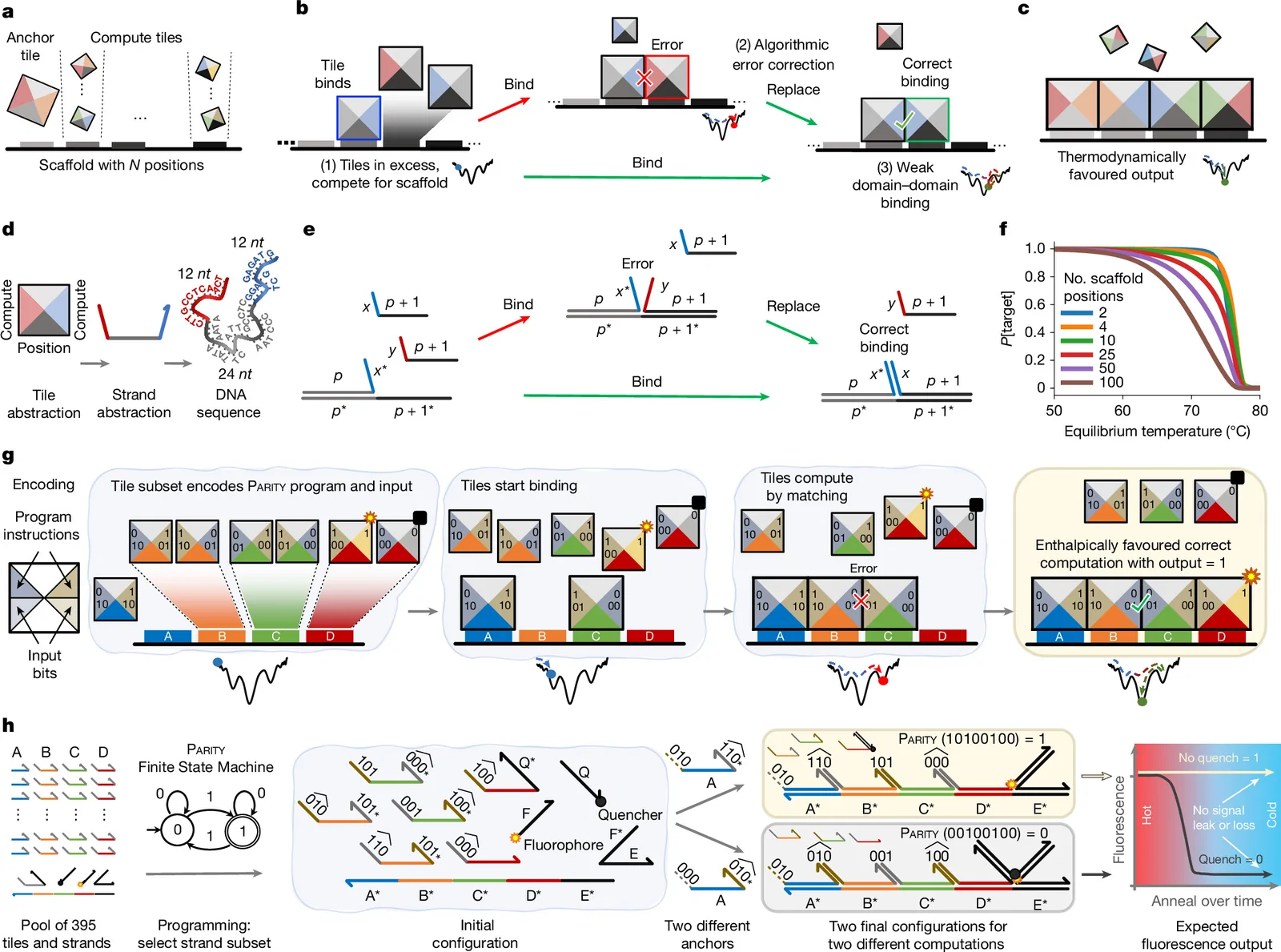
Scaffolded DNA Computer (SDC) design principles. **a**, Reprogrammable SDC architecture: scaffold with *N* positions; each position has a set of competing tiles with compute domains on left and right sides. **b**, Key concepts: (1) Tiles at high excess concentration over the scaffold, ensuring each position gets a tile. (2) Errors (mismatching colours) have an enthalpic penalty that leads to their correction by replacement. (3) Weak-affinity domains, to minimize unintended interactions such as off-scaffold assembly. **c**, From an easily prepared start state, the computation eventually leads to an enthalpically favoured fully bound target configuration. **d**, Abstraction levels. **e**, Strand-level abstraction of bind and replace primitives. **f**, Probability of target configuration at equilibrium for a given temperature using a partition function (PF) algorithm^29^. **g**, Compute domains encode program instructions (top) and input bits (bottom). Parity program computes whether an 8-bit input 10100100 has an odd or even number of 1s using program/parity bits 0 or 1 (top) and four input bit-pairs (10, 10, 01, 00; bottom). **h**, Reprogrammability by choosing strands from a large pool. Two Parity examples emphasize that output depends on every bit of input (orange, input 10100100 reports output 1; grey, input 00100100 reports output 0). Right, intended fluorescence readout cartoon, with no signal loss.

An SDC has a one-dimensional scaffold with *N* unique binding domains called ‘scaffold positions’ (Fig. 2a). A large set of tiles with some number *ℓ* of program and data bits on their left and right sides form a programming language, here *ℓ* = 3. A specific program and input correspond to a subset of tiles mixed with the scaffold. During a computation, compute tiles bind by their bottom position domain to a colour-matched scaffold position. Information is processed by the binding of adjacent tiles by colour-matched left and right sides, or compute domains. Mismatching adjacent compute domains are an algorithmic error, implying an energetic cost that will be resolved by the future replacement of one or both tiles (Fig. 2b). This molecular process of choosing the correct tile per position executes the computation, leading to a final target configuration with all colours matching. To enforce a single (deterministic) target, a program has only one tile, the anchor, for the first scaffold position (A).

Figure 2g shows an example Parity program that computes whether the number of 1s in an 8-bit input is odd or even. There are 2^8^ = 256 possible input strings, each corresponding to a different subset of seven tiles. Each compute domain encodes two input bits and a program (parity) bit. The Parity program outputs a single bit (1 for odd, 0 for even), but in general programs output up to *N* bits along the scaffold, for example, Addition below. Supplementary Note 4 expands on SDC computational theory and explains why mixing input and program bits on the same tile is a computationally legitimate encoding.

Figure 2b shows three key principles yielding the target configuration (output) being energetically favourable: (1) compute tiles are at concentration excess (typically 10×) over scaffold, to ensure every scaffold position gets a tile; (2) correct compute domain bindings are enthalpically favoured over algorithmic errors; and (3) domain-binding strength is weak enough to allow reversible binding kinetics for error correction and to prevent off-scaffold interactions. Domain-based minimum free energy (MFE) and partition function algorithms^29^ show the target having arbitrarily high probability over all other scaffolded configurations (Fig. 2f and Supplementary Note 3.5.1), using energetics estimated from our designed DNA sequences. Mathematically, the system scales well: domain strengths and lengths need only be logarithmic in *N* (ref. ^30^) (Supplementary Note 3.5.2). The SDC model meets the abstract goal in Fig. 1b.

At the more concrete strand-level of abstraction, the scaffold strand has *N* + 1 position domains (one extra domain for reporting), and each compute strand has two 12-base compute domains flanking a 24-base position domain (Fig. 2d). Figure 2e shows the tile bind operation as hybridization of the compute strand to a scaffold position and any adjacent matching compute domains. The replace operation replaces one compute strand with another. We intentionally leave the detailed DNA base-level kinetic details unspecified; we expect there to be many plausible, temperature-dependent pathways.

Under bind/replace kinetics, the expected computation time is upper-bounded by *N*^2^ for typical systems, the time for the leftmost compute domain mismatch (error) to disappear by a random walk to scaffold position *N* (ref. ^30^) (Supplementary Note 3.3). However, although we defined one reasonable kinetic model, we intentionally do not experimentally enforce any particular kinetics, a laissez-faire approach that stands in contrast to previous DNA computing methods in which precise domain-level and even base-level kinetic pathways are prescribed. We proposed that the combination of thermodynamic favourability and multiple plausible kinetic pathways would enable a straightforward temperature anneal to overcome kinetic traps, in contrast to previous use of optimized temperature holds^13–15,18–20,22,24,31^.

To avoid unintended hairpin formation within compute strands, each 3-bit sequence has two distinct compute domain sequences. A quenched-fluorescence reporting mechanism was designed to operate at any scaffold position, using 20-base domains in which low signal means output 0 and high means 1 (Methods and Extended Data Fig. 4).

## SDC programming for Addition

**Fig. 3:**
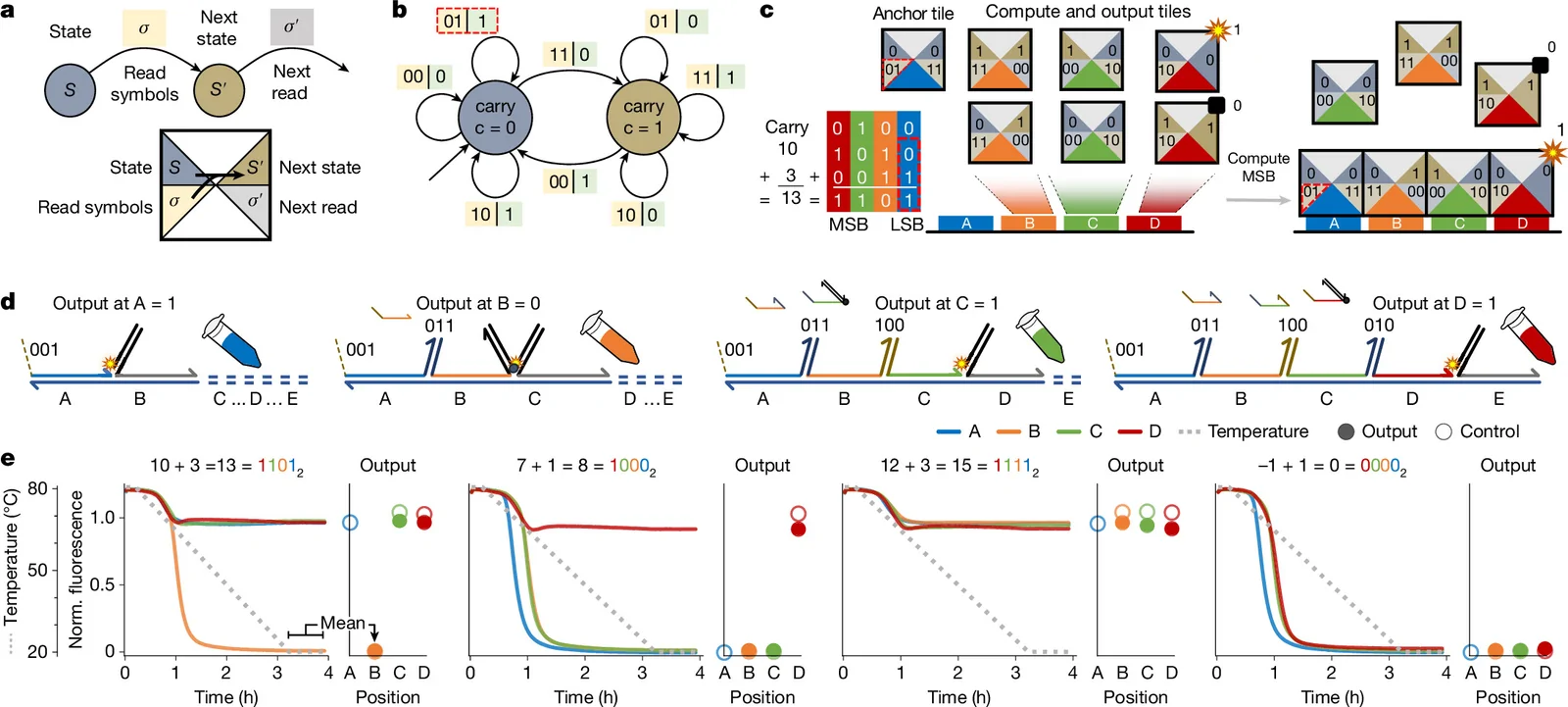
Programming and implementation of the Addition FSM. **a**, General compilation scheme from FSM into tiles; each compute domain encodes an FSM state and transition. **b**, The Addition FSM adds two binary numbers *x* + *y* = *z*: each transition reads two bits, *x*_i_ and *y*_i_, writes output bit *z*_i_ and enters carry state *c*_i_ ∈ {0, 1}. The Addition FSM is compiled to an SDC with two tiles per scaffold position. **c**, Example: 10 + 3 = 13, using colour to illustrate how bit positions map to scaffold positions. The target configuration is the most favourable as it is the only configuration with zero mismatches. **d**, Strand diagrams for reporting at each of positions A (output bit = 1), B (= 0), C (= 1), and D (= 1). Unused positions are covered with a single-domain complementary strand. **e**, Experimental results for 10 + 3 = 13, as well as three other Addition experiments. In each case, the traces show signal with respect to time and temperature, and the dot plot shows completion level as mean of 22 data points at 20 °C. The anchor position A is always non-competitive, and thus only a control. Source data

Any finite state machine (FSM)—an important sub-class of computer programs—can be compiled into a 1D SDC (Fig. 3a and Supplementary Note 4.1). The *N* = 4, *ℓ* = 3 SDC implements any 2-state 8-bit input FSM, such as 4-bit Addition (Fig. 3b). For Addition, each compute domain stores two input bits *x*_*i*_, *y*_*i*_, and a carry *c*_*i*_ computed, by tile or strand choice from the previous carry and input pair. The four output bits are each a mod 2 sum of the previous carry and input pair and were read in four separate experiments as shown in Figs. 2h and 3d and Supplementary Note 5.2.

To test the hypothesis that thermodynamic favourability facilitates simple experiments: a single-pot typical anneal dropped from 80 °C to 20 °C in 3 h, with scaffold at 100 nM concentration, then held for 45 min (Supplementary Note 7.1). Flat levels suggested a lack of kinetic traps and no signal loss (leak) (see Fig. 3e and analysis below; see the Methods for data processing). Controls have one strand per scaffold position; therefore, no per-position competition and no computation. These controls should simply assemble the ideal target configuration.

We used controls to assess SDC performance. All typical-anneal computations on *N* = 4 systems were linearly separable: completion levels were closer to their target mean control than off-target. Quantitative yield estimates were defined using the distance of the completion level to either the mean of all or to a specific high or low control (Methods). Typical anneals for *N* = 4 Addition had mean 96.7% (standard deviation (s.d.) = 0.027) yield (the single worst Addition output bit gave 91.6%), with mean 95.3% (s.d. = 0.035); overall, 10 *N* = 4 programs are reported here.

## SDC programming for nine more algorithms

**Fig. 4:**
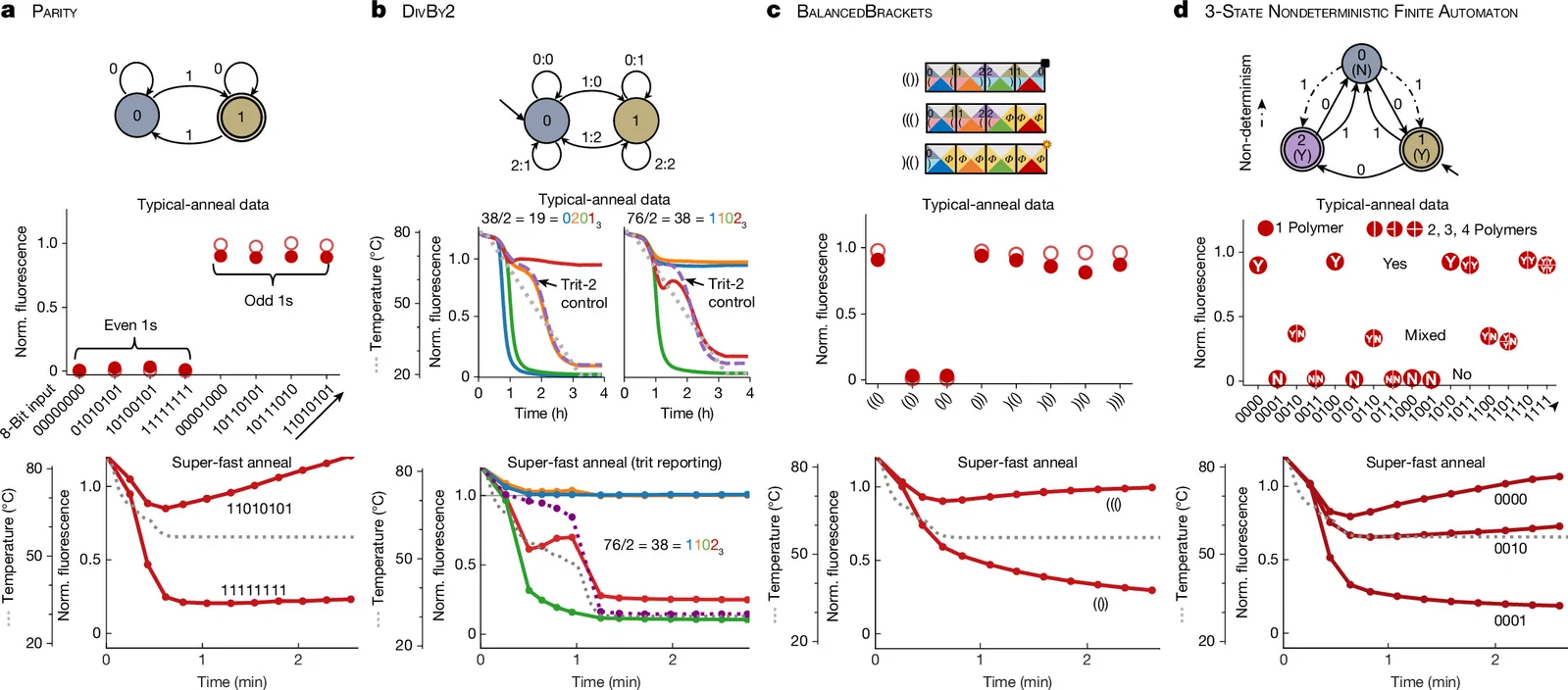
Multiple SDC programs (scaffold size *N* = 4) and super-fast anneals. More programs and details are provided in Extended Data Fig. 1. **a**, Parity program: 8-bit binary input (2 input bits per compute domain for *N* = 4 scaffold), outputs 1 if the number of 1s is odd and outputs 0 otherwise. Top, abstract program specification as FSM, which is compiled to tiles. Middle, typical-anneal completion levels. Bottom, super-fast anneals, showing dynamics. **b**, DivBy2 divides a 4-digit base-3 number by 2, giving output in base 3 (ternary). Ternary output ‘2’ is reported using a new temperature-dependent spurious-quenching system and should roughly track the trit-2 control in dashed purple (that is, the orange curve in 38/2 = 19 (left), red in 76/2 = 38 (right)). A custom trit-reporting super-fast anneal reports within a minute. **c**, BalancedBrackets: accepts balanced brackets, for example, (()), and rejects unbalanced brackets, for example, ())): by incrementing for an open bracket and decrementing for a matching close bracket, quenching only if the count is 0 at *D*. **d**, 3-State Nondeterministic Finite Automaton: each 4-bit input causes some fraction of Yes/No outputs (giving different ratios of target structures). Source data

**Extended Data Fig. 1:**
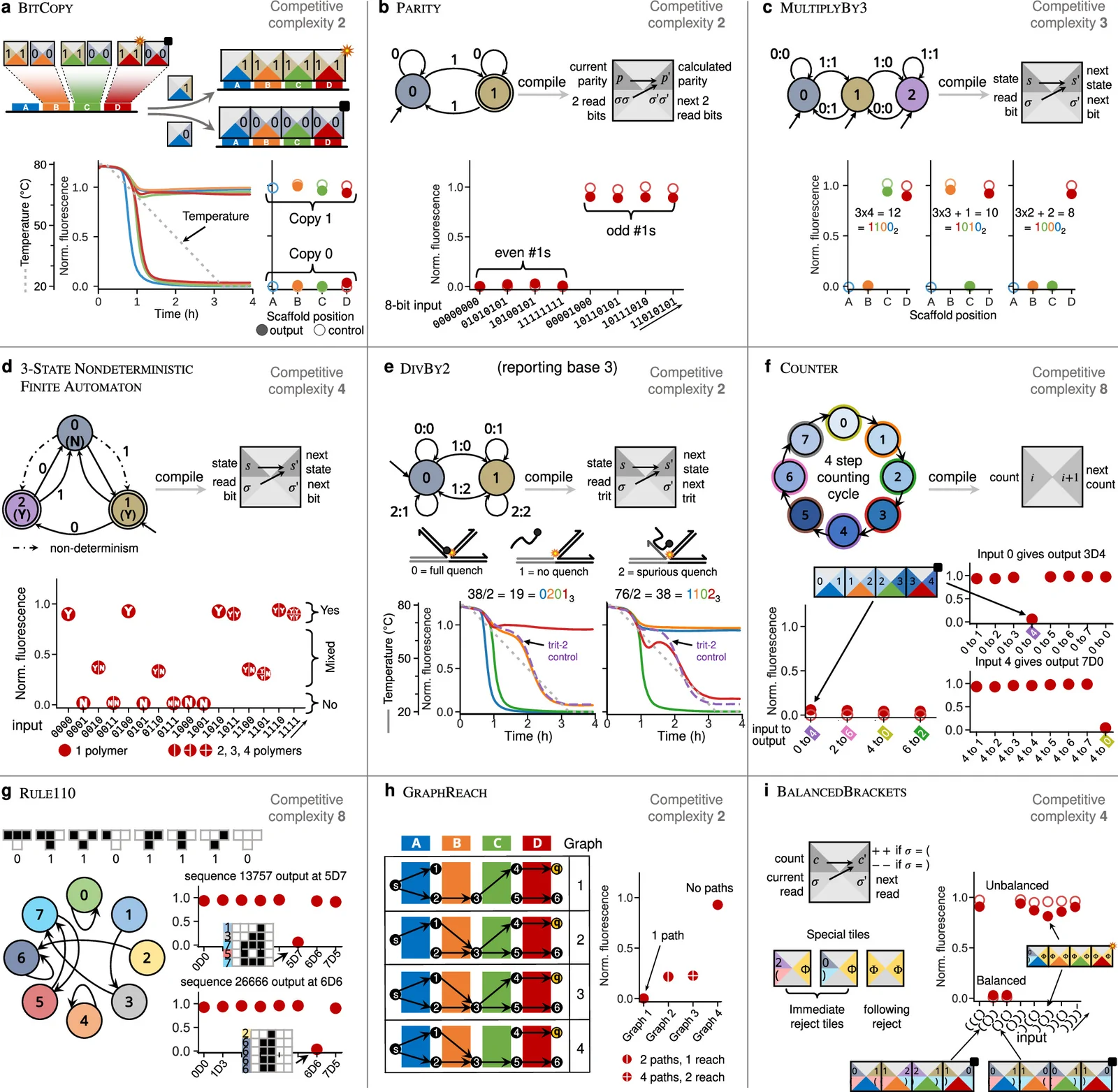
Programming 10 SDC programs of increasing complexity. **a**. Simple BitCopy program that acts as a length-4 wire; 8 traces show copying of input 0 or 1, to positions *A*, *B*, *C* or *D*. **b**. Parity program: 8-bit binary input (2 input bits per compute domain), outputs 1 if the number of ones is odd and outputs 0 otherwise. **c**. MultiplyBy3, multiplies a base 2 number by 3. **d**. 3-state Nondeterministic Finite Automaton: each 4-bit input causes some fraction of Yes/No outputs (giving different ratios of target structures). **e**. DivBy2 divides a 4-digit base-3 number by 2, giving output in base 3 (ternary) using a novel temperature-dependent spurious-quenching system. Ternary output ‘2’ should roughly track the *trit-2 control* in dashed-purple (i.e. orange curve in 38/2 = 19 (left), red in 76/2 = 38 (right)). **f**. Given a value *x* ∈ {0,1…,7} in binary, Counter counts *x* + 4 steps (e.g. input 2 at *A* yields output 6 at *D*). **g**. Rule110 simulates 4 steps of a 3-bit instance of the cellular automaton Rule 110 (with boundary condition 0 on the sides). **h**. GraphReach: finding if there is a path from start vertex *s* to goal/quench vertex *q* by forming a target structure for each path from *s*. **i**. BalancedBrackets: accepts balanced brackets, e.g. (()) and rejects unbalanced brackets, e.g. ())): increments for an open bracket and decrements for a matching close bracket, quenching only if the count is 0 at *D*. Source data

We sought to test additional hypotheses about the *N* = 4 SDC. First, we tested programmability on a further nine programs (Fig. 4 and Extended Data Fig. 1). The simple BitCopy system acts as a length-4 wire. We programmed four FSMs, which were automatically compiled to tile and strand sets from our stock, Extended Data Fig. 1b–e, including the Parity program from Fig. 2. So far, programs have two competing tiles per position (B, C, D), or competitive complexity 2. The MultiplyBy3 program multiplies by 3 in base 2, testing competitive complexity 3; average yields for competitive complexity 2 and 3 are similar: 96.2% (s.d. = 0.033) and 95.3% (s.d. = 0.041), respectively. 3-State Nondeterministic Finite Automaton tests competitive complexity 4, nondeterministically assembling up to four distinct target structures in parallel reported using distinct signal levels (Supplementary Note 7.3.5). DivBy2 divides by 2 in base 3, requiring ternary digit reporting. We used our usual scheme for reading 0 and 1, and a new, serendipitously discovered but carefully characterized temperature-dependent spurious-quenching technique for reading ternary digit 2 (Supplementary Note 5.2.2). Extended Data Fig. 1 tests programs with competitive complexity up to 8, without substantial signal loss: average yield is 95.1% (s.d. = 0.019), see Supplementary Note 7.3 for analysis. Finally, a 20-h post-anneal temperature hold showed neither leak nor slow completion (Supplementary Fig. 27), in line with theory.

## Fast SDC programs

**Extended Data Fig. 2:**
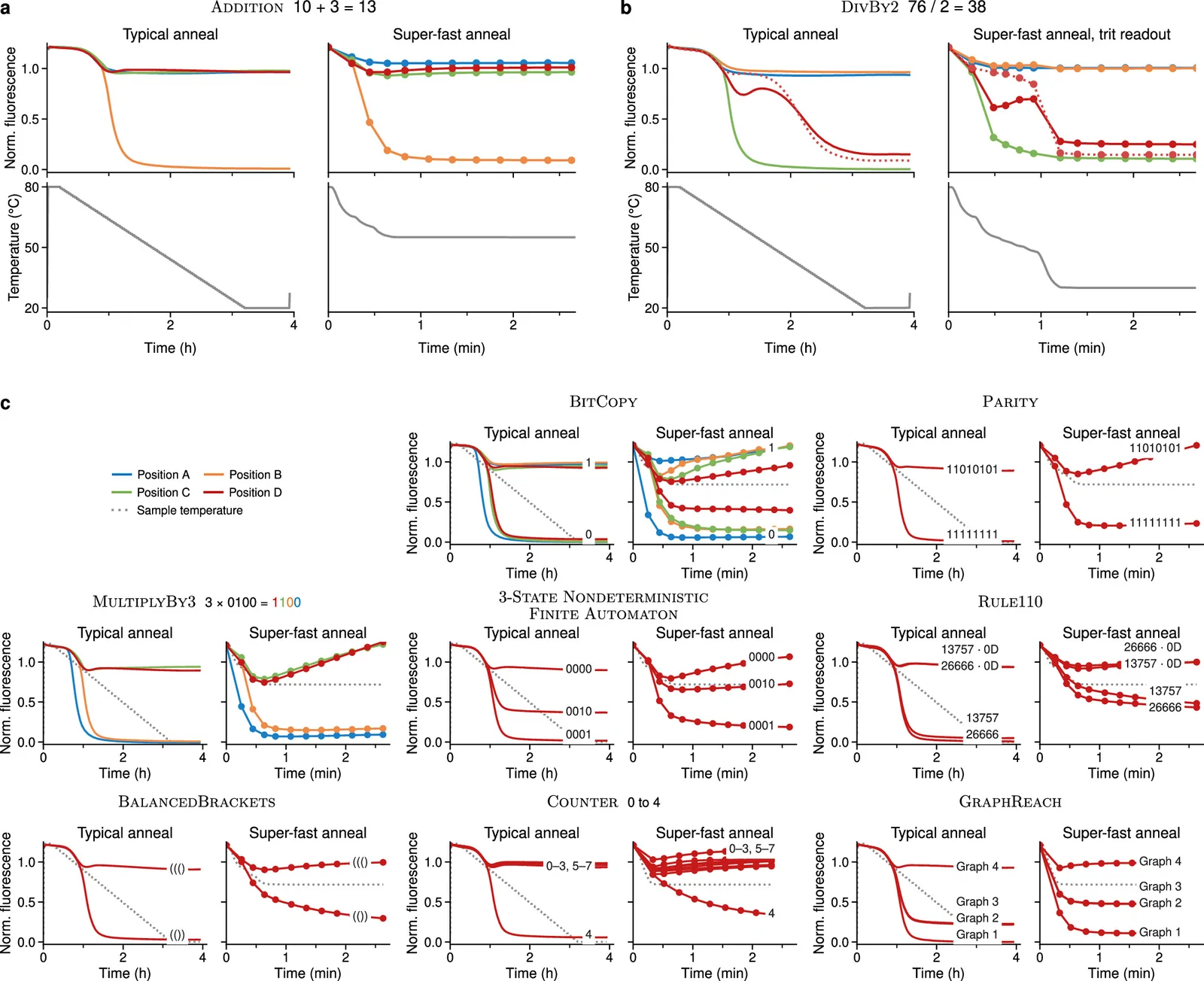
Super-fast and typical anneal computations. Computations on a variety of *N* ≤ 4 programs, using super-fast anneals. For comparison, both typical anneals (a few hours) and super-fast anneals are shown (a few minutes). Super-fast anneals generally report the correct answer in under 30 s or up to a minute for output in ternary (base 3, or trits). **a**. Addition example with *typical anneal* results on top-left, and annealing schedule (protocol) on bottom-right. Top-right shows data for the super-fast annealing schedule on bottom-right, showing clear 4-bit output in under half a minute. **b**. DivBy2 used a different annealing schedule designed to facilitate trit readouts, see Extended Data Fig. 4c. **c** Typical anneal and super-fast anneal results for selected inputs of eight other programs; Counter and GraphReach use slightly faster super-fast anneals. See Supplementary Note 7.3 for details on each program, and more results. Source data

We proposed that fast computations might be possible for short scaffold lengths, *N* ≤ 4. Super-fast anneals, dropping from 80 °C to 55 °C in under 1 min resulted in clear separation of outputs 0 and 1 (Fig. 4, Extended Data Fig. 2 and Supplementary Note 7.2.5). Results demonstrate that the SDC can compute as fast as 1 min, or even half a minute, the fastest non-trivial programs in the DNA computing literature and at the speed limit of our lab equipment. In terms of our yield performance metric, super-fast anneals have a mean 82.4% (s.d. = 0.116) yield at 1 min for Addition, and a mean of 81.2% (s.d. = 0.16) over all computations. Outputs are linearly separable with a per-program threshold (Supplementary Note 7.2.6).

## Renewable SDC computations

**Fig. 5:**
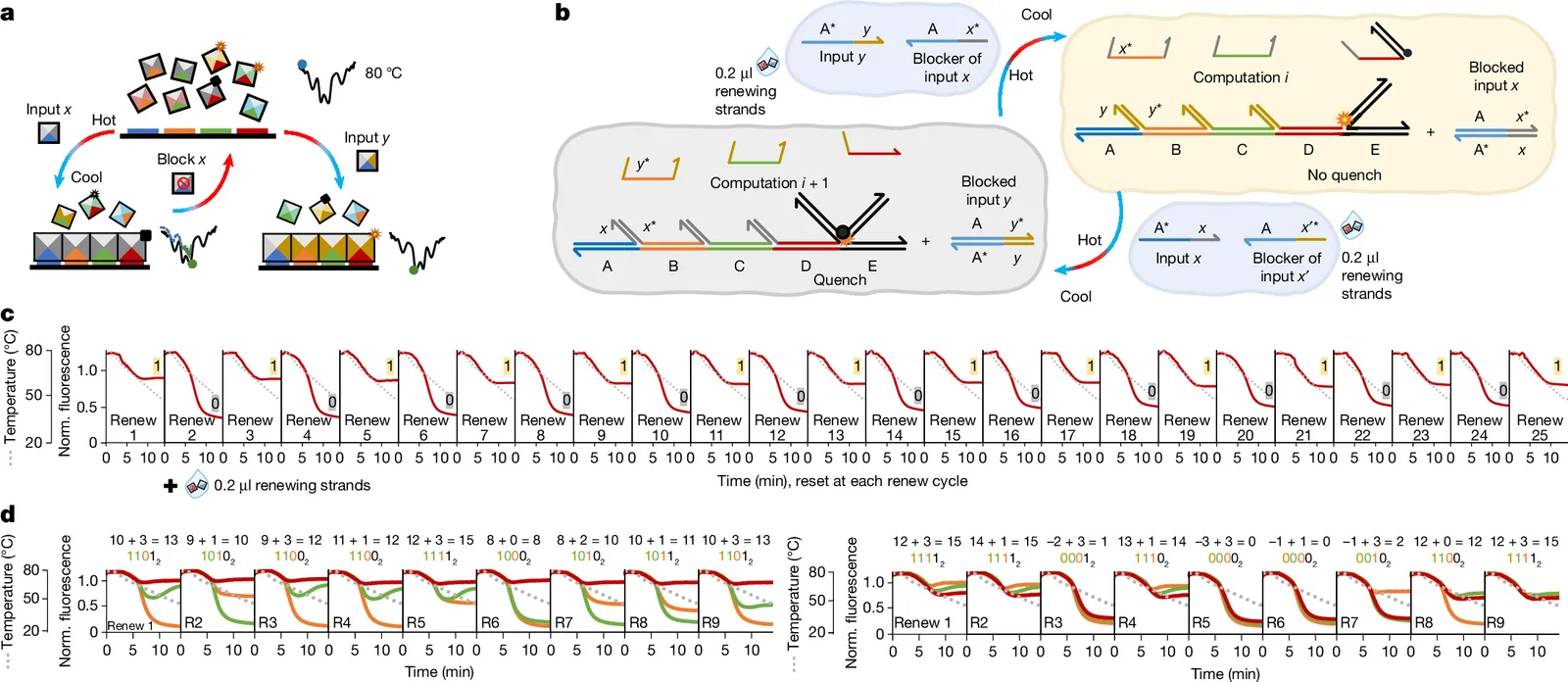
Renewable SDC programs. **a**, Principles for renewable SDC programs: at each cycle, to switch the target equilibrium we add new input *x*′ and blocker (complement) of old input *x* and reanneal to quickly reach the new target equilibrium. **b**, Strand-level renewable design: at each cycle add two strands and reanneal. **c**, Renewable BitCopy data: 25 runs, starting with input bit *x* = 1, repeatedly cycling between input bit 0 and 1 as in **b**: the blocker and new input were added at about 5.7× over scaffold (1× = 100 nM) in a 0.2 µl droplet at each renewing cycle (0 min). The sample was reannealed from 80 °C to 48 °C in 12 min (holding at 80 °C and 48 °C for 1 min (shown), and a 3–15 min gap (not shown) for inter-renew sample handling). **d**, Two renewable Addition programs: to facilitate renewing a large number (8) of distinct inputs at position A, the programs have eight tiles competing at B, reporting at three positions (B, C and D); see Supplementary Note 8.2. See Extended Data Fig. 3 for 24 runs of a renewable Counter program. Source data

We proposed that our method of programming SDC equilibria could facilitate renewable programs: simply switch equilibrium by adding a few strands that swap old input *x* for new *x*′ (Fig. 5a,b). The number of renews is limited only by the concentration decrease per re-run, which can be made arbitrarily small in the limit of large initial volume, small renew volume or low concentrations.

**Extended Data Fig. 3:**
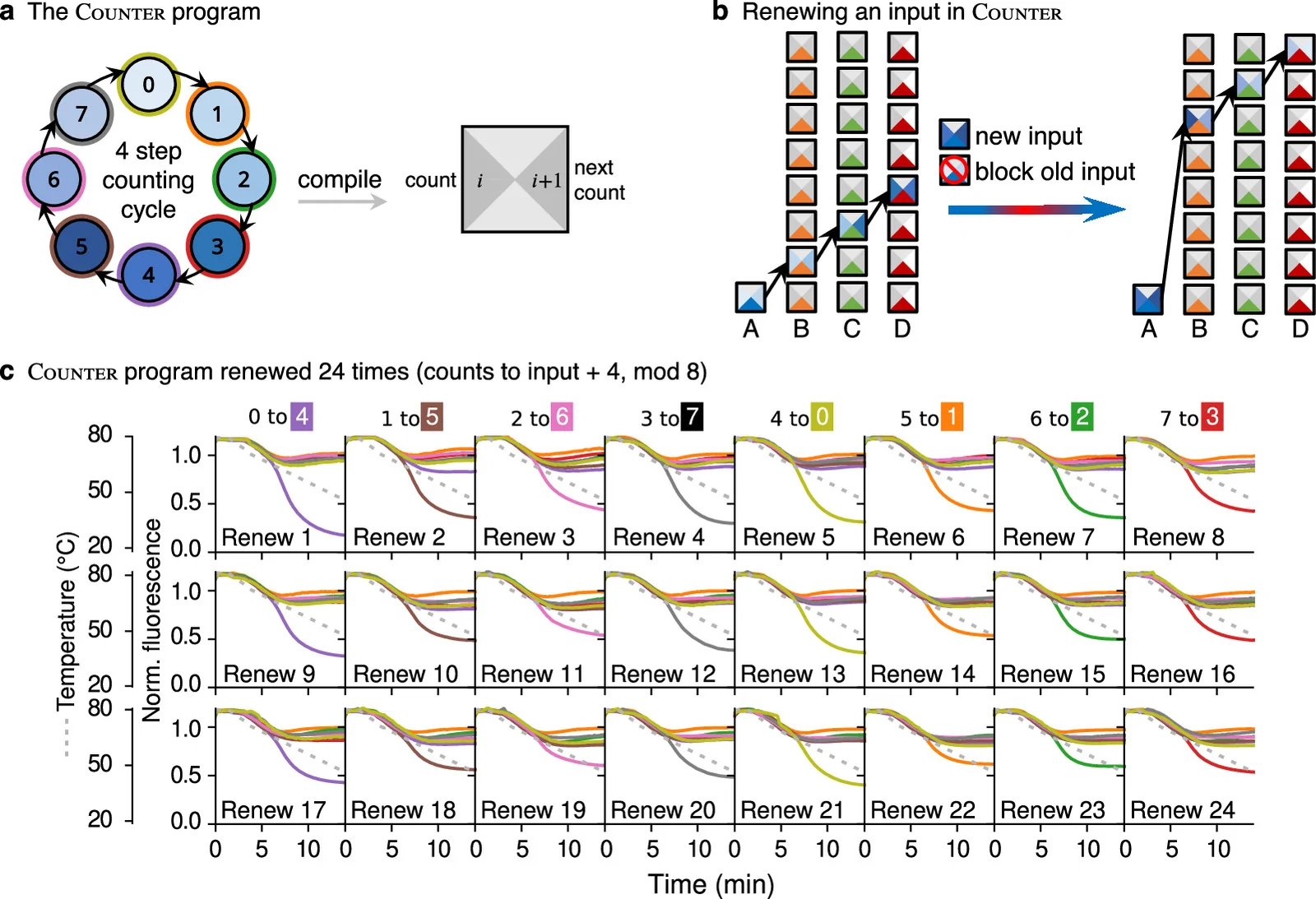
Renewable Counter program. **a**. Renewable Counter program that counts from input *x* to output *x* + 4, mod 8. Although conceptually simple, Counter has high competitive complexity with 8 tiles competing at scaffold positions *B*,*C*,*D* for base-8 counting. **b**. Schematic of the logic underlying the Counter tile program, and renewing by adding a new input and blocking the old input. Here, the height of a tile represents a number 0,1,2,3,…,7. **c**. Data showing 24 runs of Counter with different inputs. Each plot shows 8 samples, each sample ran 24 times: row 1 had 8 distinct inputs *x* ∈ {0,1,…,7} in each of the 8 samples, then row 2 and 3 repeat those 8 inputs in order (192 traces total). Source data

**Extended Data Fig. 4:**
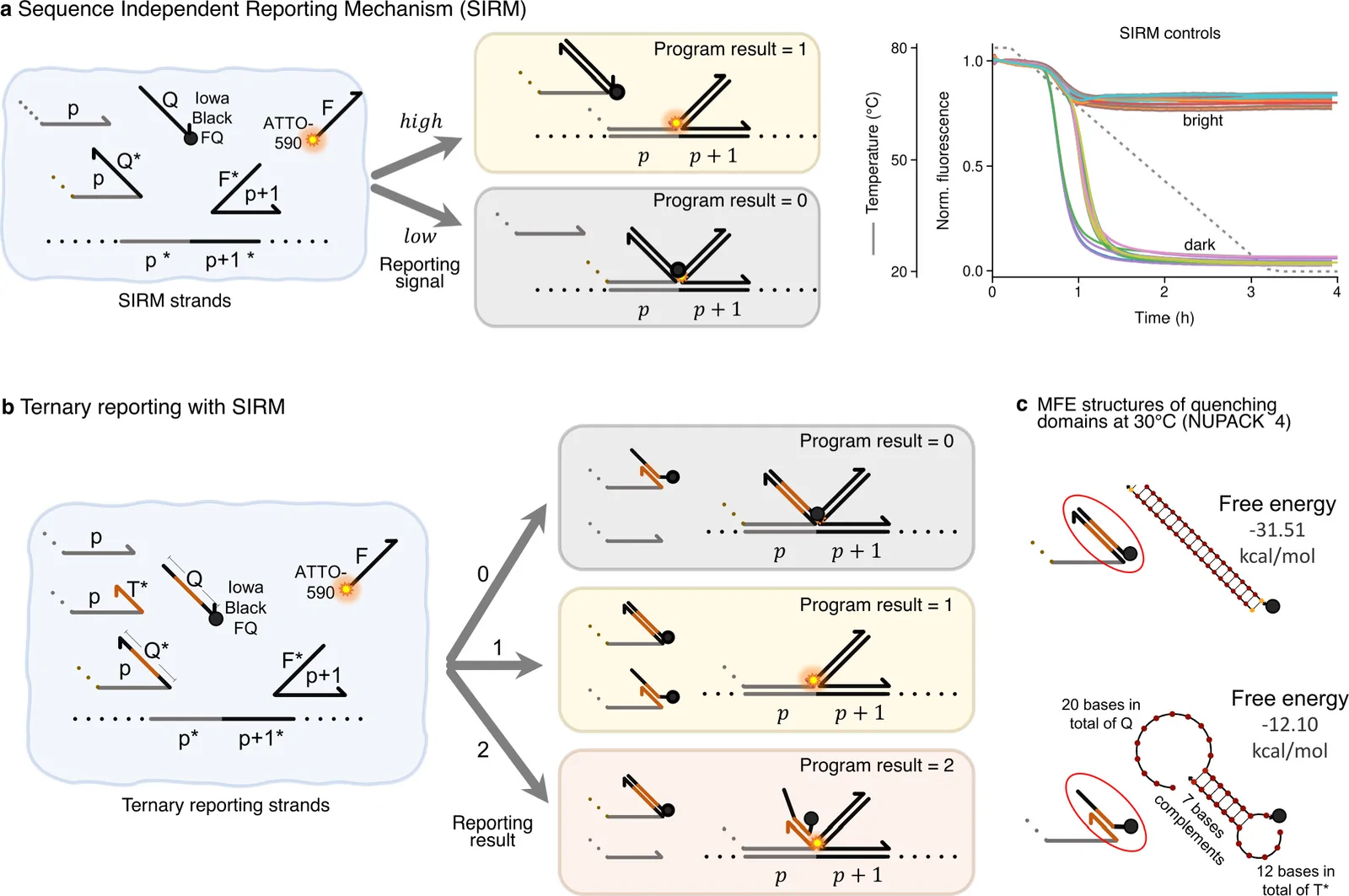
Sequence Independent Reporting Mechanism used for assaying SDC outputs. **a**. Strands used to report the output at position *p*, showing high (bright) and low (dark) fluorescence signals. A high signal occurs when the output of the computation is a strand at position *p* without a quenching domain; the quencher-carrying strand is therefore not bound to the scaffold. A low signal occurs when the output strand at position *p* has a quenching domain. **b**. Reporting mechanism for the ternary/base 3/trit values 0, 1, and 2 used in Fig. 4b of the main text. The reporting of 0 and 1 follows the mechanism shown in panel a, whereas the reporting of 2 uses partial complementarity with the quencher labelled as illustrated in panel c in more detail. **c**. NUPACK4-predicted^51^ MFE structures at 30 °C. The top structure corresponds to reporting trit 0, and the bottom structure to reporting trit 2—we serendipitously discovered this partial binding between the 12-base compute domain 5 (also called 101) and our 20-base quencher strand.

Figure 5c shows a simple BitCopy program renewed 25 times, flipping between input bit-1 and 0. Each renewal added 0.2 µl volume containing two strands using an acoustic liquid handler. For convenience, we ran fast 12 min anneals, lowering completion levels relative to typical 3-h anneals. However, the 0/1 signals were clearly separated. Renewals showed remarkably low signal degradation over time (we expected signal loss due to dilution, buildup of strands/errors distorting the intended target equilibrium and insufficient equilibration time). We sought to renew more complex programs. Two Addition programs were renewed nine times (Fig. 5d); each had eight tiles that compete at position B, allowing eight distinct inputs at position A per program (Supplementary Note 8.2). Finally, to evaluate a high competitive complexity program on a large number of renewals, we renewed Counter on 24 successive inputs (Extended Data Fig. 3). Despite eight tiles per non-anchor scaffold position, and somewhat fast anneals, all 24 renews gave a clear output, with mean signal degradation of 0.094 (s.d. = 0.033) in normalized fluorescence every eight repeats (Supplementary Note 8.2.4).

## SDC scale-up

It is tempting to interpret fast kinetics as evidence that scale-up to *N* > 4 scaffold positions is achievable. If the SDC actually obeys the simplified bind/replace model, computations would take time merely bounded by *N*^2^ (Methods). However, theory and naive optimism need tempering by some practical challenges.

We designed a rather ambitious and harsh scale-up test. Our choice of biologically synthesized 7.2 kb M13 meant having long and easily obtainable scaffold, but brought interesting non-idealities to the SDC model: (1) scaffold domains have widely varying free-energies; (2) to save material, experiments used 10 nM scaffold concentration, 10× lower than before, lowering the tile on-rate and worsening signal-to-noise; and (3) potential unintended interactions from about 6.6 kb of superfluous single-stranded M13 DNA. Moreover, all except the two labelled strands were unpurified, to save time and cost.

**Extended Data Fig. 5:**
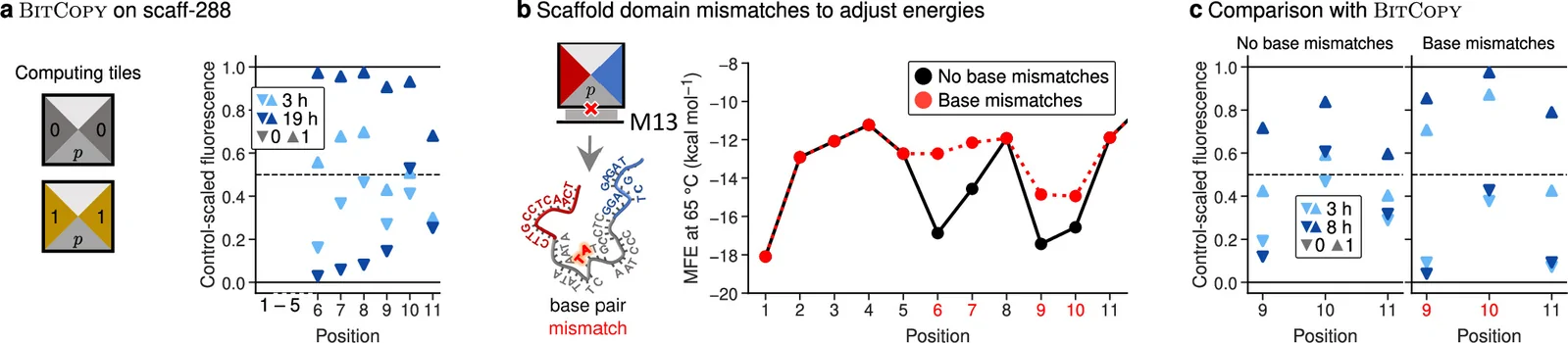
Scaffold energies and mismatches. **a**. Scale-up of BitCopy program to an 11-domain scaffold (scaff-288): yields are improved by longer anneal times, but yields falter by size 10. **b**. As scaff−288 is derived from M13, its associated scaffold domains have significantly varying binding energies. One option for adjusting them to be more isoenergetic is to add base pair mismatches to the scaffold domains of tiles for stronger positions. **c**. Introduction of mismatches moderately improves yields. Source data

An initial test on an 11-position M13 scaffold subsequence suggested that uneven scaffold domain-binding energetics would be a challenge. DNA base mismatches improved signal, but not enough for our ambitious scale-up plans (Methods and Extended Data Fig. 5). To achieve further scale-up and improve kinetics, we used thermodynamic sequence design principles to choose a good 624-base M13 segment (Supplementary Note 9.1). But even our best choice, scaff-624, had large variability in scaffold domain duplex Δ*G*° of −20.9 kcal mol^−1^ to −11.1 kcal mol^−1^ at 65 °C, compared with designed compute domains (−9.42 kcal mol^−1^ to −6.61 kcal mol^−1^).

**Fig. 6:**
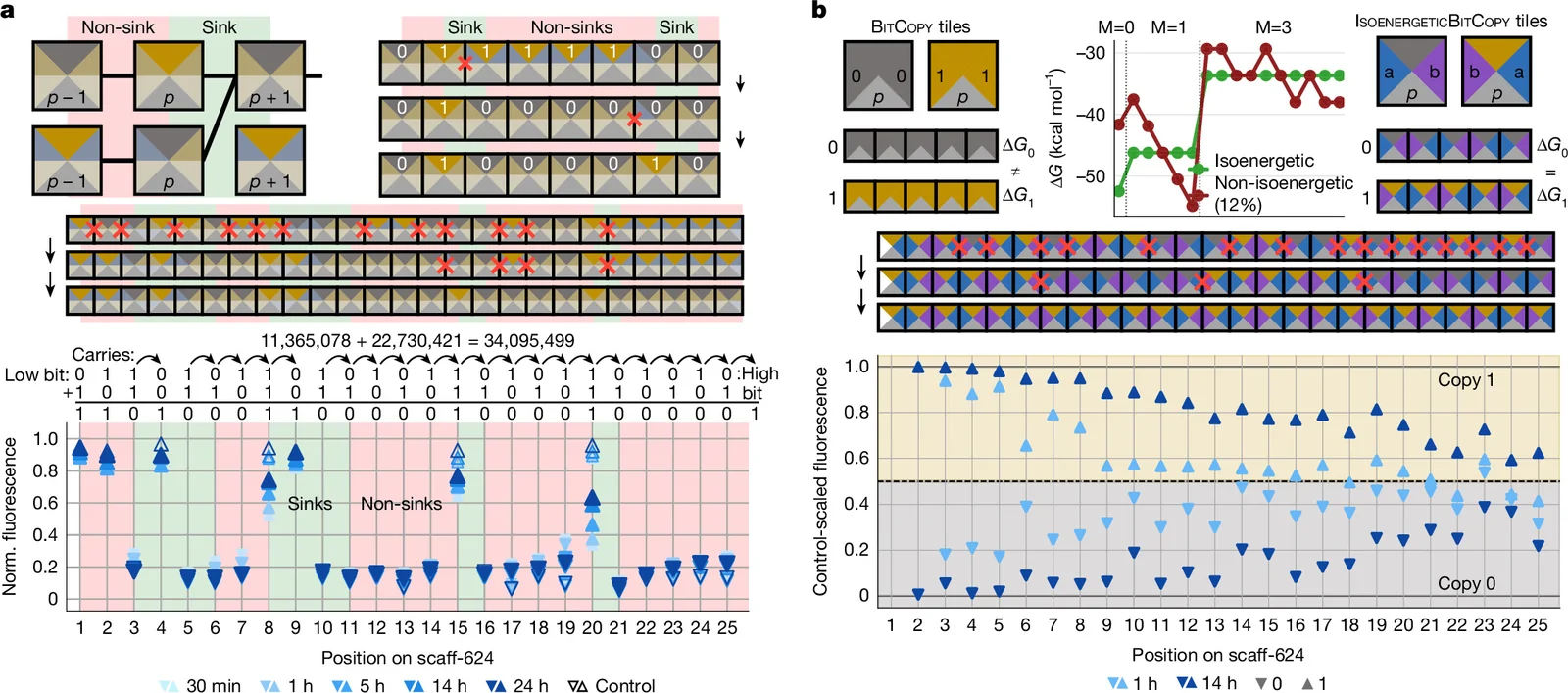
Energy landscape programming for SDC scale-up to 25 positions. **a**, Scale-up of the Addition program to a 25-domain scaffold (scaff-624). The program logic uses sinks and non-sinks to provide explicit error correction by absorbing carry bits in green-shaded regions, which truncates error pathways in the energy landscape. Data show Addition of 2 × 25-bit numbers, including 25 carry and 25 output bits, giving 100 bits of compute. **b**, In the BitCopy program (left), because 0 and 1 compute domains may have different binding strengths (non-isoenergetic), a configuration with errors (compute domain mismatches) may be erroneously more thermodynamically favourable than one without (middle)^30^. IsoenergeticBitCopy rearranges compute domains such that two competing tiles have the same domains but in a different order, which, in turn, implies that configurations with a fixed number of mismatches are approximately isoenergetic yielding a nice energy landscape (green) without unintended traps, a form of energy landscaping applicable to any SDC program^30^. Data show reliable 25-position computation in 14 h. Source data

Computational theory suggested two approaches for more systematic programming of the energy landscape. First, SDC tile programs may embed logic with good error-reduction properties. Each Addition position is either a sink that absorbs a 0/1 carry bit or a non-sink that passes the carry along—non-sinks are more susceptible to logical bit-flip errors because of increased tile competition. For a pair of inputs, let *M* be the longest run of non-sinks. Random Addition inputs have a low mean *M* of 4.0 (s.d. = 1.6), implying a logical structure conducive to error suppression. An *M* = 4 computation is computed in roughly 1 h (Supplementary Note 9.4). A harder *M* = 5 example in Fig. 6a has good completion within 14 h. *M* ≤ 5 covers more than 84% of pairs of 25-bit numbers; hence, most of the *M*-hardness of Addition. Supplementary Note 9.4 includes harder computations.

Second, we developed a programming-based method for isoenergetics: the IsoenergeticBitCopy program target configurations for inputs 0 and 1 are almost isoenergetic, because both reuse the same compute domains, respectively, encoding 0101 … 01 and 1010 … 10. This form of compute domain relabelling can be systematically applied to any SDC program with scale-up^30^, enforcing configurations with *k* compute domain mismatches to sit on a roughly flat energy plateau (Fig. 6b and Supplementary Note 9.5). This gave excellent results, copying a bit across 20 positions with 71% yield, or 25 positions with 59% yield, on a 14 h anneal (Methods). DNA base mismatches did not further improve yield significantly (Supplementary Note 9.5).

## Discussion

The concept of thermodynamic favourability (Fig. 1) enabled robust, fast, renewable and scalable molecular computing. The core concept was elucidated on 10 SDC programs and over 700 computations with up to 50-bit binary input and 100 bits of compute, as well as base-3 strings and graphs. Unlike out-of-equilibrium molecular computers, the SDC did not need explicit error correction by redundancy or scale-up^13,15,32–36^, nor compute-strand purification, precisely calibrated temperature holds^13–15,33,34^, multi-step manual protocols^18–20,22–24^ or early experiment termination to avoid leak. The SDC is concentration-robust, inheriting the beautiful principle from DNA origami^27^ of having compute or staple strands in large concentration excess over the scaffold. We even repeated one experiment after 1.5 years, adding water as its 96-well plate had partially dried out, giving the data reported for MultiplyBy3 and Parity.

Intuition suggests equilibrium computation could be imprecise, because a desired output configuration competes with exponentially many off-target structures. We outline thermodynamic and kinetic arguments against this intuition. Our thermodynamic design principles engineer the predicted target (MFE) structure to have probability approaching 1.0, driving the sum of probabilities of all other structures to 0 (Fig. 2f and Supplementary Note 3.5.1). Notably, we saw correct program outputs with average completion and yield on typical anneals, at scaffold length ≤4, of 95.3% (s.d. = 0.035) of strict controls with fewer strands and simpler equilibria. Mean yield at scaffold positions B and C was 98.1% (s.d. = 0.014), dropping to 94.4% (s.d. = 0.034) at D. Increasing competitive complexity from 2 to 8 did not significantly degrade yield for typical 3-h anneals.

For larger *N*, mere thermodynamic favourability may not be sufficient: we need a well-shaped, traversable energy landscape. The abstract SDC tile model gives a stepped landscape, efficiently explorable by random walks that ratchet forward by compute domain mismatch repair. Hence, in theory and practice, we took a somewhat liberal approach to kinetics. The bind/replace model in Fig. 2b and Supplementary Note 3.3 begins a computation by guessing one of a large set of $O({2}^{N})$ initial states, yet by mere random walk completes in only ≤*N*^2^ time^30^. However, experimentally, we did not enforce this particular kinetics, nor any other: we simply annealed the system, suggesting that something akin to bind/replace is happening at higher temperatures. We used a strand design without intentional kinetic traps (Fig. 2), but did not carry out design optimizations, nor did we seek to characterize and prevent off-pathway interactions, approaches common in typical non-equilibrium molecular computing^13,15,18–20,22,24,33,34^. Our approach is perhaps comparable to that of DNA origami^27^, in which assembly kinetics is not precisely controlled, yet the design works beautifully^37–39^, although further scaling may require kinetic design^30^. Super-fast 1-min anneals ran at the speed limit of our laboratory equipment and beat previous speed records for non-trivial DNA computations^26,40–42^. Results for our super-fast and typical anneals indicate that the SDC has fast, high-temperature, reversible assembly kinetics (Supplementary Note 3.6). Scaling-up to scaffold length 25 systems required slower anneals. The awkward energetics of cheap biologically derived scaffold (M13) slowed computation at some positions, but in a way that seems not unlike our proposed random-walk kinetics (Supplementary Note 3.3). We conclude that the SDC is robust by design despite much remaining to be understood.

Although the earliest DNA motor was reusable^43^, designing renewable molecular computers is an important challenge because of system complexity and out-of-equilibrium operation^21,44–50^. Here, three SDC programs were renewed 9, 24 or 25 times at only 12 min per cycle (plus time to add inputs). Previous work demonstrated 16 (ref. ^21^), 6 (ref. ^47^), 3 (refs. ^44,45^) and 2 renewals (refs. ^25,46^) (that is, changing input; note that resetting the same input or computation is permitted in many systems^13,14,21,25,26,33,34,49^). Some systems require manual purification steps^21,44–47^ or molecular technology beyond DNA^48,50^. The principle of thermodynamic favourability^25^ seems to provide a rather simple and effective method for program renewal.

Thermodynamic computing principles could enable robust DNA data storage^20^ or endow DNA origami^27^ with the computational ability to algorithmically drive nanostructure formation. Robustness properties could facilitate thermodynamic computing in complex biological environments, engineered in RNA or protein. We used a biologically sourced scaffold sequence as a proof of principle for under-designed DNA sequences.

Our work shows that DNA systems are amenable to ‘free-energy landscaping’, enabling equilibrium molecular computing to move from theory to practice. Related theoretical work^29^ gives strategies for further scaling the SDC through choice of domain energies, isoenergetic programming and analysis^30^. The thermodynamic binding network model^3^ gives another principled approach, recently demonstrated^25^ on entropy-driven equilibrium programs (Supplementary Note 2.3). And equilibrium computing by strand commutation encodes Boolean circuits as a cascade of weakly bound, base-mismatching strands^26^. Fuel-consuming, infinite-time, out-of-equilibrium molecular dynamics^10,17,43^ seem, by definition, unsuited to thermodynamically favoured computation. However, even there, energy landscapes can be sculpted to encode complex, finite-time dynamics while driving to a ground state—similar to a fixed-count chemical oscillator^17^, for example. Beyond molecular programming, the theory of thermodynamics of computation^2–4,9,10^ seeks to determine ultimate energetic costs for computation. The SDC has several obvious costs (for example, number and length of strands, heating and annealing), and optimizations^30^ are warranted for further scale-up or applications such as data storage or generalizing to higher-dimensional structures. It will be interesting to see how these challenges develop in comparison to other equilibrium and non-equilibrium computing architectures, be they molecular^13,15,32–36^, classical digital-electronic^2,4,9^ or quantum^11^. With this work, we hope to stimulate dialogue^7^ towards consideration of thermodynamically favoured, energy-efficient computing platforms.

## Methods

### Sequence design

For scaff-120, we intentionally chose not to use de novo designed DNA sequences for the scaffold for ease of sourcing long scaffolds and to prototype a system that is robust to sequence choice, even biologically sourced. We chose to have four scaffold position domains (A, B, C and D), and the additional reporting position E, of 24 bases each giving a 120-base scaffold sequence, scaff-120, arbitrarily chosen to be two contiguous subsequences of M13 (Supplementary Note 6). For scaff-288, we used a contiguous subsequence of biologically sourced M13, instead of a shorter synthetic scaffold; consisting of four 24-base domain sequences from scaff-120 (B, C, D and E) plus eight more 24-base contiguous domains; enabling computations on 11 scaffold positions (Supplementary Note 9.3). For scaff-624, we also used the biologically sourced full M13 molecule in solution, but chose a 624-base contiguous subsequence (26 domains) based on the criteria given in Supplementary Note 9. We used a thermodynamic sequence design approach^13^ for compute and reporting domains.

For the compute domains, we used a three-letter ATC code with at most one G as an exception, motifs CCCC, GGGG, AAAAA and TTTTT were forbidden, and isoenergetic complementary binding interactions of −11.7 ± 0.1 kcal mol^−1^ (chosen simply by our metric). The value −11.6 is the computed mean for a pool of 10,000 length 12 sequences. As soft constraints (that is, enforced with potential exceptions), orthogonal interactions (unintended binding) >−2 kcal mol^−1^ were evaluated using binding(A,B) = pfunc(A,B) - pfunc(A) - pfunc(B) as in Supplementary Information section 4.2.1 and p. 43 of ref. ^13^, with pfunc() given by NUPACK4^51^. Secondary structure within each sequence compute domain was optimized to be >−0.25 kcal mol^−1^. Reporter domains were designed using similar principles (isoenergetic binding between −21.7 kcal mol^−1^ and −21.95 kcal mol^−1^ at 53 °C and no G was permitted to be within four bases of a fluorophore^52,53^). Software packages nuad^54^ and NUPACK4^51^ were used for DNA sequence design.

### Strand design

To have a system that is programmable, we designed a set of compute domains that can be used interchangeably to create an expressive programming language of compute tiles and strands. To avoid unintended hairpin formation within compute strands, each three-bit sequence has two distinct compute domains (for example, 000 and $\hat{000}$, meaning they have DNA sequences that are not complementary; Supplementary Note 5.1), giving 2 × 2^3^ = 16 distinct compute domains for *ℓ* = 3. A quenched-fluorescence reporting mechanism, operating at any scaffold position was designed. By convention, quenched (low) signals report output bit 0 and unquenched (high) signals bit 1. Fluorescence and quencher-labelled strands are 20 bases long.

The *N* = 4 scaffold positions and *ℓ *= 3 bits per compute domain imply 201 compute strands: a 120-base scaffold (named scaff-120), 64 strands at each position B, C and D, and 8 strands at anchor position A (unpurified). An additional 64 strands for reporting (purified) and 130 for renewable programs (unpurified), give a pool of 395 strands for *N* ≤ 4, plus an additional 1,144 for *N* ≤ 25 (Supplementary Note 5.5). We used a thermodynamics-based sequence design approach for compute and reporting domains, building on previous work^13^, with details in Supplementary Note 6.

### DNA synthesis and fluorophore–quencher labelling

Apart from the biologically sourced M13 scaffold strand, which was ordered from tilibit, the other 1,539 DNA strands used in the system were ordered from Integrated DNA Technologies (IDT). Non-scaffold strands were generally ordered in 384-well or 96-well plates normalized to 200 µM in IDTE pH 8.0 buffer. All strands were ordered unpurified except for seven strands used in scaff-120 experiments: the 120-base synthetic scaffold (Ultramer DNA Oligo PAGE purified and dry; Supplementary Note 7.7), the two fluorophore–quencher-labelled strands (HPLC purified and normalized to 100 µM in IDTE pH 8.0 buffer), the four unlabelled reporting-related strands that bind to both scaff-120 and fluorophore-labelled strands (PAGE purified and normalized to 100 µM in IDTE pH 8.0 buffer). We used ATTO590 as a fluorophore label and Iowa Black FQ from IDT as quencher.

### Sample mixing and buffer conditions

Most experimental mixes were generated using the custom cosmix library^55^, and some using the riverine library^56^. SDC programs and controls were mixed using an Echo 525 acoustic liquid handler (Beckman Coulter, supplied by Labplan Ltd. Ireland) for the data reported in Figs. 3–6, and by hand for some of the data in the Supplementary Information. Also, earlier versions of some data in Figs. 3–5 were mixed by single- and multi-channel hand pipettes, meaning that a liquid handler is not necessary to run SDC programs, but we found that it gave more consistent results (lower signal variance) than hand-mixing. Picklists specifying the liquid transfer sequence and volumes from the 384-well source plates to the 96-well destination plates were generated using the above-mentioned Python libraries. Mixes using synthetic scaff-120 were prepared with a scaffold concentration of 1× = 100 nM, whereas scaff-288 and scaff-624 used biologically sourced M13 at 1× = 10 nM concentration. That is, in all experiments, 1× is the scaffold concentration. All systems used 10× compute strands (except renewable programs used 5.7×), 0.93× of the reporting-related strands that bind to both the fluorophore-labelled strand and scaffold (for example, ATTO*B, ATTO*C, ATTO*D, ATTO*E), 0.79× of the fluorophore strand (5RF) and 17.86× of the quencher strand (3RQ); see beginning of Supplementary Note 10 for domain–strand naming conventions. DNA strands for an SDC program were placed in a single 0.1 ml 96-well PCR plate (or tube) to a volume of 35 µl in 12.5 mM Mg^++^ in Tris-acetate-EDTA (TAE) buffer with 0.01 % Tween.

### Fluorescence spectroscopy and temperature protocols

Bulk fluorescence data were run in a quantitative 96-well PCR machine (QuantStudio 5 Real-Time PCR System operated using the open-source Python library qslib^57^) for fluorescence measurement over time. All experiments used the ATTO590 fluorophore with excitation at 593 nm and emission at 622 nm. Seven plates were used to run the experiments of the 10 programs shown in Figs. 3 and 4 and Extended Data Fig. 1, including controls and repeats. Anneals essentially consisted of dropping temperature from 80 °C to 20 °C over durations ranging from 30 s to 24 h, then holding at 20 °C, at which completion levels are computed (Supplementary Note 7.1). Often, temperature is dropped at a quicker rate in the 75–55 °C range than in the 55–20 °C range. All temperature protocols are available; see section ‘Data and Code availability’ below.

It should be noted that our models would predict that an anneal (of, for example, BitCopy) would go from high signal (around 1.0) at 80 °C down to around to 0.5 (that is, reporting a random output bit at high temperature), and then level off at the completion level of around 0.0 or 1.0 as the target configuration is computed. The reason we do not see an intermediate (0.5) high-temperature signal is that our 20-base labelled or reporting strands are binding below that temperature. Future work may make use of longer reporter domains (data not shown).

### Data analysis and normalization

Data were minimally processed: (1) each raw fluorescence trace was normalized by dividing by the mean of its readings at 80 °C; (2) traces were averaged across at least two repeats; and (3) for scaff-120, traces were rescaled so that the mean completion levels of 0- and 1-reporting controls were 0 and 1.0, respectively (Supplementary Notes 7.2.3 and 7.2.4). The rescaling step was not done for scaled-up systems, in which individual control completion levels are reported instead (see below). The performance metrics calculations are discussed in Supplementary Notes 7.2.3, 7.2.4 and 9.2.

### SDC performance

We estimated SDC performance in three ways: the qualitative separation of output bit-0 from bit-1 and the two quantitative metrics that seek to estimate the proportion of correctly assembled target structures. First, qualitatively, data for all typical-anneal computations on *N* = 4 systems (4 scaffold domains) are linearly separable: all samples have completion levels closer to their target mean control level than off-target. Second, our main quantitative yield estimation is the difference between a given experimental sample completion level (actually the mean of a pair of technical repeats), which should be high (respectively, low), and the mean of all high (respectively, low) controls. This is the number reported as yield for all *N* = 4 experiments and is called metric 1 in Supplementary Note 7.2.6. Finally, metric 2 in Supplementary Note 7.2.6 simply compares a given experimental sample completion level with that of the control of the sample (usually the absolute difference between the mean of two technical repeats of the control and the mean of two technical repeats of the experiment); this metric was used for *N* > 4 (scaled-up) systems that use an M13 scaffold because of its widely varying domain-binding energetics. Supplementary Note 7.2.6 summarizes yields for all programs tested.

### DNA base mismatches

In seeking to scale up to larger values of *N*, we initially tested an *N* = 11 BitCopy system, on a 288 nucleotide (nt) M13 region called scaff-288, chosen for backwards compatibility with scaff-120. Yields were worse than for the *N* = 4 case, but increasing anneal time from 3 h to 14 h improved results. To combat poor M13 scaffold domain energetics, we used DNA base mismatches^58,59^, giving a clear yield improvement from 39% to 57% on 11 positions over an 8 h anneal (Extended Data Fig. 5 and Supplementary Notes 9.2 and 9.3). However, these results and widely varying scaffold domain energetics suggested more careful scaffold sequence selection was warranted for system scale-up, leading to extensive DNA sequence design and analysis of M13 to choose a better M13 segment; see Supplementary Note 9.1 (which still has fairly poor energetics compared with what our theoretical and simulation models would suggest as being more optimal^30^).

## Online content

Any methods, additional references, Nature Portfolio reporting summaries, source data, extended data, supplementary information, acknowledgements, peer review information; details of author contributions and competing interests; and statements of data and code availability are available at https://doi.org/10.1038/s41586-026-10996-5.

## Supplementary information

Supplementary Information This file contains Supplementary Information sections 1–10 and Supplementary References.

Peer Review File

## Source data

Source Data Fig. 3

Source Data Fig. 4

Source Data Fig. 5

Source Data Fig. 6

Source Data Extended Data Fig. 1

Source Data Extended Data Fig. 2

Source Data Extended Data Fig. 3

Source Data Extended Data Fig. 5

## Data Availability

Python code and libraries for sequence design, echo mixing automation, qPCR protocols, and data analysis and processing are available as supplementary files to this submission at Zenodo^60^ (https://doi.org/10.5281/zenodo.15869377). Source data are provided with this paper.

## References

[1] Simmel, F. C. in *Visions of DNA Nanotechnology at 40 for the Next 40: A Tribute to Nadrian C. Seeman* (eds Jonoska, N. & Winfree, E*.*) 17–29 (Springer, 2023).

[2] Wolpert, D. H. The stochastic thermodynamics of computation. *J. Phys. A Math. Theor.* **52**, 193001 (2019).

[3] Doty, D., Rogers, T. A., Soloveichik, D., Thachuk, C. & Woods, D. Thermodynamic binding networks. In *Proc.**DNA23: The 23rd International Conference on DNA Computing and Molecular Programming* Vol. 10467, 249–266 (Springer, 2017).

[4] Bennett, C. H. The thermodynamics of computation—a review. *Int. J. Theor. Phys.* **21**, 905–940 (1982).

[5] Du, Y. & Mordatch, I. Implicit generation and modeling with energy based models. In *Proc. Advances in Neural Information Processing Systems* Vol. 32 (eds Wallach, H. et al.) (Curran Associates, 2019).

[6] Kirkpatrick, S., Gelatt, C. D. Jr & Vecchi, M. P. Optimization by simulated annealing. *Science* **220**, 671–680 (1983). 17813860 10.1126/science.220.4598.671

[7] Frank, M. P. & Conte, T. M. Reversible computing technology is essential for sustainable growth of the digital economy. Preprint at www.sandia.gov/app/uploads/sites/210/2022/06/FrankConte-HotCarbon22-v4SAND.pdf (2022).

[8] Demaine, E. D., Lynch, J. F., Mirano, S. & Tyagi, A. Energy-efficient algorithms. In *Proc. ACM Conference on Innovations in Theoretical Computer Science* 321–332 (ACM, 2016).

[9] Landauer, R. Irreversibility and heat generation in the computing process. *IBM J. Res. Dev.* **5**, 183–191 (1961).

[10] Ouldridge, T. E. The importance of thermodynamics for molecular systems, and the importance of molecular systems for thermodynamics. *Nat. Comput.* **17**, 3–29 (2018). 29576756 10.1007/s11047-017-9646-x PMC5856891

[11] King, A. D. et al. Quantum critical dynamics in a 5,000-qubit programmable spin glass. *Nature* **617**, 61–66 (2023). 37076625 10.1038/s41586-023-05867-2

[12] Adleman, L. M. Molecular computation of solutions to combinatorial problems. *Science* **266**, 1021–1024 (1994). 7973651 10.1126/science.7973651

[13] Woods, D. et al. Diverse and robust molecular algorithms using reprogrammable DNA self-assembly. *Nature* **567**, 366–372 (2019). 30894725 10.1038/s41586-019-1014-9

[14] Evans, C. G., O’Brien, J., Winfree, E. & Murugan, A. Pattern recognition in the nucleation kinetics of non-equilibrium self-assembly. *Nature* **625**, 500–507 (2024). 38233621 10.1038/s41586-023-06890-z PMC10794147

[15] Schulman, R., Yurke, B. & Winfree, E. Robust self-replication of combinatorial information via crystal growth and scission. *Proc. Natl Acad. Sci. USA* **109**, 6405–6410 (2012). 22493232 10.1073/pnas.1117813109 PMC3340064

[16] Fern, J. & Schulman, R. Design and characterization of DNA strand-displacement circuits in serum-supplemented cell medium. *ACS Synth. Biol.* **6**, 1774–1783 (2017). 28558208 10.1021/acssynbio.7b00105

[17] Srinivas, N., Parkin, J., Seelig, G., Winfree, E. & Soloveichik, D. Enzyme-free nucleic acid dynamical systems. *Science* **358**, eaal2052 (2017). 29242317 10.1126/science.aal2052

[18] Bui, H. et al. Localized DNA hybridization chain reactions on DNA origami. *ACS Nano* **12**, 1146–1155 (2018). 29357217 10.1021/acsnano.7b06699

[19] Chatterjee, G., Dalchau, N., Muscat, R. A., Phillips, A. & Seelig, G. A spatially localized architecture for fast and modular DNA computing. *Nat. Nanotechnol.* **12**, 920–927 (2017). 28737747 10.1038/nnano.2017.127

[20] Wang, B., Wang, S. S., Chalk, C., Ellington, A. D. & Soloveichik, D. Parallel molecular computation on digital data stored in DNA. *Proc. Natl Acad. Sci. USA* **120**, e2217330120 (2023). 37669382 10.1073/pnas.2217330120 PMC10500265

[21] Song, T. & Qian, L. Heat-rechargeable computation in DNA logic circuits and neural networks. *Nature* **646**, 315–322 (2025). 41034583 10.1038/s41586-025-09570-2 PMC12507696

[22] Qian, L. & Winfree, E. Scaling up digital circuit computation with DNA strand displacement cascades. *Science* **332**, 1196–1201 (2011). 21636773 10.1126/science.1200520

[23] Wickham, S. F. J. et al. A DNA-based molecular motor that can navigate a network of tracks. *Nat. Nanotechnol.* **7**, 169–173 (2012). 22266636 10.1038/nnano.2011.253

[24] Thubagere, A. J. et al. A cargo-sorting DNA robot. *Science* **357**, eaan6558 (2017). 28912216 10.1126/science.aan6558

[25] Wang, B., Chalk, C., Doty, D. & Soloveichik, D. Molecular computation at equilibrium via programmable entropy. *Sci. Adv.* **12**, eadx3969 (2026). 41512058 10.1126/sciadv.adx3969 PMC12787524

[26] Nikitin, M. P. Non-complementary strand commutation as a fundamental alternative for information processing by DNA and gene regulation. *Nat. Chem.* **15**, 70–82 (2023). 36604607 10.1038/s41557-022-01111-y

[27] Rothemund, P. W. K. Folding DNA to create nanoscale shapes and patterns. *Nature* **440**, 297–302 (2006). 16541064 10.1038/nature04586

[28] Castro, C. E. et al. A primer to scaffolded DNA origami. *Nat. Methods* **8**, 221–229 (2011). 21358626 10.1038/nmeth.1570

[29] Shalaby, A., Thachuk, C. & Woods, D. Minimum free energy, partition function and kinetics simulation algorithms for a multistranded Scaffolded DNA Computer. In* Proc. 29th International Conference on DNA Computing and Molecular Programming (DNA 29)* Vol. 276, 1:1–1:22 (Schloss Dagstuhl, 2023).

[30] Petrack, J., Evans, C. G., Cervera Roldan, A., Enayati, M. & Woods, D. Scaling up thermodynamically favoured scaffolded DNA computing by sculpting the energy landscape. In *Proc. 32nd International Conference on DNA Computing and Molecular Programming (DNA32)* Vol. 387*,* 7:1–7:22 (Schloss Dagstuhl, 2026).

[31] Thachuk, C., Winfree, E. & Soloveichik, D. Leakless DNA strand displacement systems. In *Proc. 21st International Conference on DNA Computing and Molecular Programming* (eds Phillips, A. & Yin, P.) Vol. 9211, 133–153 (Springer, 2015).

[32] Winfree, E. & Bekbolatov, R. Proofreading tile sets: error correction for algorithmic self-assembly. In *Proc.**9th International Workshop on DNA Based Computers* (eds Chen, J. & Reif, J.) Vol. 2943, 126–144 (Springer, 2004).

[33] Evans, C. G. Crystals that count! Physical principles and experimental investigations of DNA tile self-assembly. PhD thesis, Caltech (2014).

[34] Schulman, R., Wright, C. & Winfree, E. Increasing redundancy exponentially reduces error rates during algorithmic self-assembly. *ACS Nano* **9**, 5760–5771 (2015). 25965580 10.1021/nn507493s

[35] Wang, B., Thachuk, C., Ellington, A. D., Winfree, E. & Soloveichik, D. Effective design principles for leakless strand displacement systems. *Proc. Natl Acad. Sci. USA* **115**, E12182–E12191 (2018). 30545914 10.1073/pnas.1806859115 PMC6310779

[36] Wang, B., Thachuk, C. & Soloveichik, D. Speed and correctness guarantees for programmable enthalpy-neutral DNA reactions. *ACS Synth. Biol.* **12**, 993–1006 (2023). 37014808 10.1021/acssynbio.2c00356 PMC10127273

[37] Lee Tin Wah, J., David, C., Rudiuk, S., Baigl, D. & Estevez-Torres, A. Observing and controlling the folding pathway of DNA origami at the nanoscale. *ACS Nano* **10**, 1978–1987 (2016). 26795025 10.1021/acsnano.5b05972

[38] Dunn, K. E. et al. Guiding the folding pathway of DNA origami. *Nature* **525**, 82–86 (2015). 26287459 10.1038/nature14860

[39] Rossi-Gendron, C. et al. Isothermal self-assembly of multicomponent and evolutive DNA nanostructures. *Nat. Nanotechnol.* **18**, 1311–1318 (2023). 37524905 10.1038/s41565-023-01468-2 PMC10656289

[40] Zhu, Y. et al. Accelerating DNA computing via freeze-thaw cycling. *Sci. Adv.* **9**, eaax7983 (2023). 37624882 10.1126/sciadv.aax7983 PMC10456841

[41] Kennedy, T., Pearce, C. & Thachuk, C. Fast and robust strand displacement cascades via systematic design strategies. In *Proc.**28th International Conference on DNA Computing and Molecular Programming (DNA 28)* Vol. 238, 1:1–1:17 (Schloss Dagstuhl, 2022).

[42] Song, T. et al. Fast and compact DNA logic circuits based on single-stranded gates using strand-displacing polymerase. *Nat. Nanotechnol.* **14**, 1075–1081 (2019). 31548688 10.1038/s41565-019-0544-5

[43] Yurke, B., Turberfield, A. J., Mills, A. P. Jr, Simmel, F. C. & Neumann, J. L. A DNA-fuelled molecular machine made of DNA. *Nature* **406**, 605–608 (2000). 10949296 10.1038/35020524

[44] Liu, L. et al. Multifunctional clip strand for the regulation of DNA strand displacement and construction of complex DNA nanodevices. *ACS Nano* **15**, 11573–11584 (2021). 34213302 10.1021/acsnano.1c01763

[45] Eshra, A., Shah, S., Song, T. & Reif, J. Renewable DNA hairpin-based logic circuits. *IEEE Trans. Nanotechnol.* **18**, 252–259 (2019).

[46] Garg, S. et al. Renewable time-responsive DNA circuits. *Small* **14**, 1801470 (2018). 10.1002/smll.201801470 30022600

[47] Genot, A. J., Bath, J. & Turberfield, A. J. Reversible logic circuits made of DNA. *J. Am. Chem. Soc.* **133**, 20080–20083 (2011). 22111514 10.1021/ja208497p

[48] Song, X., Eshra, A., Dwyer, C. & Reif, J. Renewable DNA seesaw logic circuits enabled by photoregulation of toehold-mediated strand displacement. *RSC Adv.* **7**, 28130–28144 (2017).

[49] Hahn, J. & Shih, W. M. Thermal cycling of DNA devices via associative strand displacement. *Nucleic Acids Res.* **47**, 10968–10975 (2019). 31584082 10.1093/nar/gkz844 PMC6847259

[50] Li, X. et al. Enzyme-assisted waste-to-reactant transformation to engineer renewable DNA circuits. *Chem. Commun.* **55**, 11615–11618 (2019). 10.1039/c9cc05941e 31501837

[51] Fornace, M. E., Porubsky, N. J. & Pierce, N. A. A unified dynamic programming framework for the analysis of interacting nucleic acid strands: enhanced models, scalability, and speed. *ACS Synth. Biol.* **9**, 2665–2678 (2020). 32910644 10.1021/acssynbio.9b00523

[52] Padirac, A., Fujii, T. & Rondelez, Y. Quencher-free multiplexed monitoring of DNA reaction circuits. *Nucleic Acids Res.* **40**, e118 (2012). 22753028 10.1093/nar/gks621 PMC3424586

[53] You, Y., Tataurov, A. V. & Owczarzy, R. Measuring thermodynamic details of DNA hybridization using fluorescence. *Biopolymers* **95**, 472–486 (2011). 21384337 10.1002/bip.21615 PMC3082624

[54] Doty, D. & Lee, B. UC-Davis-molecular-computing/nuad. *GitHub* https://github.com/UC-Davis-molecular-computing/nuad (2022).

[55] Stérin, T. tcosmo/cosmix. *GitHub* https://github.com/tcosmo/cosmix (2022).

[56] Evans, C. G. Riverine. *Zenodo*10.5281/zenodo.6861213 (2022).

[57] Evans, C. G. cgevans/qslib: v0.11.0 (v.0.11.0). *Zenodo*10.5281/zenodo.10072151 (2023).

[58] Machinek, R. R. F., Ouldridge, T. E., Haley, N. E. C., Bath, J. & Turberfield, A. J. Programmable energy landscapes for kinetic control of DNA strand displacement. *Nat. Commun.* **5**, 5324 (2014). 25382214 10.1038/ncomms6324

[59] Olson, X. et al. Availability: a metric for nucleic acid strand displacement systems. *ACS Synth. Biol.* **6**, 84–93 (2017). 26875531 10.1021/acssynbio.5b00231 PMC5259754

[60] Stérin, T., Eshra, A., Evans, C. G., Adio, J. & Woods, D. Data and code for “A Thermodynamically Favoured Molecular Computer: Robust, Fast, Renewable, Scalable”. *Zenodo*10.5281/zenodo.15869377 (2026).42749917

