# Supplementary material for: A thermodynamically favoured molecular computer
Source: Nature. 2026 Sep 16;657(8132):646–52. doi: 10.1038/s41586-026-10996-5 (PMC13581610; doi:10.1038/s41586-026-10996-5)
Supplement: Supplementary file 2 [file 41586_2026_10996_MOESM2_ESM.pdf]

nature portfolio

Peer Review File

A Thermodynamically Favoured Molecular Computer

Corresponding Author: Professor Damien Woods

Any redactions in this file are there to maintain patient confidentiality, the confidentiality of unpublished data, or to remove third-party material.

This file contains all reviewer reports in order by version, followed by all author rebuttals in order by version.

Version 0:

Reviewer comments:

Referee #1

(Remarks to the Author)
1. Summary of the key results
This article implements the idea of computing through thermodynamic annealing using DNA strands, demonstrating the concept through multiple steps of executing a finite state machine. they show that computing can occur accurately and quickly using this implementation for a range of computations and use these results to support the notion that computing by approaching thermodynamic equilibrium should be considered more broadly

2. Assessment of novelty

It should be said in fairness that everything, on some level has been done or thought of. It is also the case that in a short form article it is impossible to mention all related work.

With that said, there are two areas of concern.

First, the article makes it sound like all computers are iterative driven processes. It is certainly true that this is the dominant computing mode. However, the notion of thermodynamic computing is in itself not new. The idea of annealing to find an answer to a computation problem is the foundation of a family of statistical techniques, most directly simulated annealing. (This process is run on a computer but it is very much in the spirit here.) A general sentence or two (or section in the SI, though I hesitate to suggest so much expansion to the incredible body of work already there) of discussion about this field would improve the paper and the context.
Further, in other areas of computing, such as quantum computing, I am not an expert but my understanding is that this notion of searching an exponentially large space to find a low energy configuration that represents an answer is a central concept there or could have central relevance in that searching through an exponential number of quantum states and preferring some due to, presumably, a lower energy state, is the fundamental advantage of quantum computing.

Second, I think a couple of fundamental points were missed with respect to other methods of DNA computing and there are a couple of missing references about similar work. Two stand out:
a. The authors state that algorithmic self-assembly is not a thermodynamic means of computation because it requires nucleation. However, that requirement for nucleation is more correctly understood as a means to efficiently compute the thermodynamic minimum by providing a kinetic pathway toward that minimum. Conceptually, this reviewer is not convinced that at equilibrium there would not be the same preference to a perfect computation (because of the need to minimize mismatches) in this process. This process would in fact directly map to Woods' beautiful recent work in Nature on algorithmic self-assembly (2019). In this, I find the claims about algorithmic self-assembly misleading.
b. There are other recent articles about using thermodynamic minimums to compute logic functions. Most notable is Nikitin, Nature Chemistry, 2023. This article also has weaknesses but the authors need to address distinctions in their approach, though it is recognized that the comprehensive treatment of the subject here is interesting. This article might also provide encouraging points about scaling of the processes studied here, which are a critical challenge about significance (described below).

3. Assessment of significance

This paper is notable in its enthusiasm and original discussion. It stimulates thought and forces one, as great science should, to think big picture and perhaps how this field (unconventional computation) might make large strides.

That said, I think that the paper's ultimate contribution is less dramatic than it might first appear. This issue is not, specifically, the challenge of novelty per se, though the issues described above are important to address.

The issue is rather the question of the ultimate benefits and power of thermodynamic computing, which do not at all deserve the treatment they deserve in such an article.

Figure 1 is a great visual and it does capture the magic of thermodynamic computing, which is nice. But it also misleads the reader, as the figure makes it seem as if there is only one error state. This is correct if computing were only one step. However, in a computing processes that is, whether iterative or a search that requires either computing space or computing steps (one or the other must grow as the complexity of a computation increases, speaking in the roughest terms), the size of this space increases exponentially and a computation must decide between one of an exponential number of trajectories.

In "driven" computing, this decision is made by a ratcheting effect that each of a set of steps is irreversible. As such each step must be decided cleanly (at the cost of energy) but the upshot is a benefit in terms of time taken to compute - it scales linearly with the number of computing steps even as the number of potential trajectories increases exponentially. The authors are correct that this costs energy.

In thermodynamic computing as the authors describe it, this exponential number of potential trajectories must all be considered at once. The energy minimum can be found, but the challenge of doing so increases exponentially with the number of computing steps in the finite state machine.

This challenge has been considered, for example in Levinthal's paradox in protein folding. In Levinthal's paradox, however, the solution is actually to return to the kinetic perspective and to observe that energy landscapes without a clear "folding funnel" are selected again, though my understanding of this area is limited. Such a "folding funnel" would, however, be exactly the kind of "pathway engineering" the authors seek to avoid here. This suggests that the problem with this idea is in its scaling -- it works beautifully with 5 steps, where there are perhaps on order 3^5 = 243 configurations, but once one goes to even 10 or 15 steps the number of trajectories that needs to be distinguished is untenable - rapidly approaching millions or billions as the complexity of the annealing process increases exponentially.

The fundamental limitation to this paper is the authors' systematic ignoring of this key fact to at least temper their discussion, which does a disservice to the field. Undoubtedly. the results are neat -- the implementation is elegant, and the experiments and results are clear and well done.

But the emphasis of the article confused me. The key question I had was "what if you did 10 or 20 computing steps"? Does this really work? This is not that hard to do -- modern synthesis could easily produce the scaffold needed for each of these processes, and the longer strand would be the only step needed. I would fine 2 demonstrations of 10 or 20 step computing process more compelling than the panel in Figure 5 which honestly prove the same points as the results in previous figures are not that surprising. Having a longer scaffold would also allow a proper study of the time needed rather than an extended discussion of maxing out the speed of the thermocycler used. The experimenters were clever but right now what their experiment suggests is clearly "less than 20 minutes" for 5 steps for a great answer and a mediocre answer in just 1-2 (which is exciting, to be clear!) but could easily be studied by looking at different 8, 10 or 12 etc step processes and different annealing speeds. If, indeed, the scaling is surprisingly great and trackable (see next paragraph), that could be something to report in Nature.

One could also argue that the number of steps is not the point -- of course it will stop working at some point. But to that I would say, first, that it is the point -- if it works well for a 50-step computation, that is vastly more exciting than working only for 5 but not for 8. If the former, it suggests that most computations we do with molecules now or wish to do for a range of biological processes, could be done easily by annealing and complex chemistries maybe aren't needed. Second, the number of steps being finite demands discussion as in now universe is this number sufficient to for example consider annealing to replace silicon or quantum computers for difficult calculations. With this in mind, how might thermodynamic computing here be visualized as a component within a bigger process? This should be addressed with at least a sentence or two in the impact section beyond "This is a conversation piece" if this is really an article for general interest, as the authors have more expertise than the general reader on what the impact is here.

4. Other comments / improvements

Line 3 of abstract: "Unlike life, decades of theory shows" -> "Decades of theoretical work shows that unlike life,"
Line 4 of abstract: "can execute" without a direct object is awkward
Line 7: remove comma after "a"
Line 42: What is fuzzy equilibria

Figure 2f : From reading the SI the x axis caption should be "temperature" and the idea is that this is the probability of being in the right configuration given that the system reached equilibrium for that temperature. The caption should be improved to clarify what is being plotted.

The authors allude to origami as a means of thermodynamic annealing that scales to a large number of strands. It is true that large DNA origami can fold well but there are several key points about that the authors leave out which really diminish the power of this metaphor to believing that this process will scale well:
1. Origami folding is highly cooperative (Dunn et al, Nature, 2015). This fact makes origami folding more efficient than the assembly here as the number of strands increases. But it also points out that origami folding explores far fewer potential configurations than this process aims to and so (as that article shows) will not necessarily find the thermodynamic minimum in a short time period.
2. Even despite the limited space of exploration, the long folding times needed for naively designed DNA origami (cf Douglas et al, Nature, 2009) can be extreme -- weeks, suggesting also that scaling here will not be favorable.

There is a bunch of what I might term "jargon" in this paper, such as CG clamps, and it doesn't generally feel written for the general reader. For these and other reasons, most importantly maximize the impact of this work, I suggest a journal such as Nature Physics rather than Nature for this article (see next section)

5. Appropriateness for this journal

This paper is exciting and refreshing in its genuine enthusiasm. Reading it over, I felt that some of its weaknesses with respect to publication in Nature might be strengths for an alternative journal choice, which would be Nature Physics.

This recommendation is in part because this paper does not represent the jump in the capability of the model over prior work -- thermodynamic computing has been done in less elegant forms before and the demonstrations here do not provide a surprising increase in the capability of the process over prior work.

It is also because, fundamentally, I disagree with the claims made in the abstract that this article will lead to reduced energy consumption. The authors' results do not support that. Thermodynamic computing costs energy because one must wait for the computation to complete and maintain specific environmental conditions to allow that to happen. The exponential increase in this time is likely to make this result of more academic interest than of any immediate practical interest. Unfortunately, the article, by skirting this fact rather than facing this real question and trying to contextualize the results rather than making a lot of a small demo, could end up shutting the door on this direction before it should be shut (and which happened, perhaps, as a result of Adleman's seminar 1994 work, which I was disappointed not to see cited here).

But I also think that appropriate placement of this paper will increase its impact over placement in Nature, where I feel it will create confusion and perhaps even backlash, neither of which is needed. The authors state, and I agree mightily, that this paper is a great piece for discussion for advancing the world of nontraditional computing. And that the need to think about new approaches to unconventional computing is also great due, yes, to the extreme challenges of energy costs.

Its SI ,which in itself has a lovely review of the field (and really should appear as a separate piece, honestly) is great context. The careful analysis of the thermodynamics should be in the article itself, as it is much more important (in my opinion) than the various demos (after 2-3) in Figure 5 in terms of advancing the work -- it is a coherent theory that is lovely and really does provide a place to move this field forward and think about how to build better molecular (and other) computers in a systematic yet creative and free way. But the way this article is structured now, this contribution is not at the center as it should be.

(Remarks on code availability)
I read some of the SI but again, in the interest of providing feedback on the significance, did not read all of us but could do so, though I would want to ask a postdoc for help to do a good job.

Referee #2

(Remarks to the Author)
In this paper the authors put forward a potentially new (and general) strategy for error free computing. This strategy is based on using the thermodynamically preferred ground state as a basis for computation. A contrast is drawn to the so called typical paradigm in which the end result of the computation is not the thermodynamic ground state but rather a metastable state.

There a lot of interesting points in this paper but I find a few key points confusing. The entire paper is seemed to be based on the premise that a large class of computation outputs can be mapped onto thermodynamic ground states. Is this always true ? Doesn't frustration due to mutiple competing pathways make computing with metastable states a possibility always ? In saying this, I am considering computation done in/using/by the Hopfield inspired models.

Another central ingredient here seems to be that the multiple "replace" pathways allow the system to effectively perform thermodynamic computation (as in reach the lowest free energy state/state with the best possible enthalpy). Again, this is a very interesting point but I am not completely sure how general this can be. Is annealing with some rate a pre-requisite ? In asking these questions, I am partially motivated by a similar class of questions that emerge when dealing with self-assembly. There, kinetic traps are known to arrest formation of complex structures. It is known that in some cases a quench in protocols/temperature might help nucleate the correct structure. Is that similar to what the authors are proposing ? Explaining this will help (in my opinion) imrprove the readability of the paper and help it reach a broader range of readers.

I am happy to re-evaluate this work after the authors answer the above questions.

(Remarks on code availability)

Version 1:

Reviewer comments:

Referee #4

(Remarks to the Author)
I think this is exciting work, in particular because of the robustness and simplicity of the system compared to existing DNA computation approaches. Having read through the prior reviewers' comments and author responses, I have no major additional points. I have mainly suggestions/questions that may help improve readability/comprehensibility for a more general scientific reader.

Fig 1F. Can the authors scale this theoretical analysis to larger numbers of scaffold positions to gain an understanding if there a practical limit arising from not being able to use a low enough equilibrium temperature (i.e. freezing conditions or kinetics too slow).

Related, comparing the error rates of Figure 6 c and d, it seems that using different compute domains across the tiles is important, as perhaps using identical compute domains for tiles at multiple positions as in BITcopy might be causing problems. In the discussion about scaling, it could be helpful to include theoretical discussion of how the diversity of compute domain sequence space may or may not impact scaling.

Some typos (did not catch all, but noticed these): line 126: "level". 213: "distinct". 304: "in". Figure 6 caption: "prevents truncates".

Fig 3e. Could the authors include a sentence or two in the methods or supplements, (I might have missed it), for why the fluorescence starts off above the normalized value of 1? Is there some basal level of quenching that occurs after an hour?

I preface this question by saying I think I am missing something conceptually about all dna-based computation, and likely is my understanding issue. But potentially other readers coming from a more general background might have a similar question: Is the fact that the physical instantiation of the computer being the same as the input strands themselves (or at least including the input strands themselves) placing DNA computation in a different category than an FSM? Taking the ADDITION example, one would select a specific set of DNA oligos corresponding to that addition. This to me seems like you are using hardware/perhaps robotics to execute an initial operation that uses an algorithm to find those oligos and mix them. Then the oligos carry out a computation that essentially handles carryovers. Where in this whole process is the computation actually occurring? Is part of it actually being carried out in advance in the selection and physical selection and mixing of oligos? And how does this compare to the execution of computation using electronic hardware which seems more autonomous and general and not something that needs to be physically morphed for each different computation. I think I understand that this is a physical implementation of a FSM, but maybe the point I'm tripped up over is that for this implementation, the FSM is changing each time: in particular, most oligos from the library are being left out.

I have a suggestion on providing more explanation in the presentation for a broader scientific audience. I personally would call myself peripherally aware of work in the DNA computing field, well informed about molecular biology, and an engineer by training, so I think I'm somewhat able to follow basic mathematical/physicist/computer scientist principles. Given that background, which I suspect would be representative of many who would be interested in this work but not steeped in computer science, I had substantial difficulty following what the "000, 000 with a bar" notations meant (I realize it is explained in the supplement but would be quite buried for a general reader), what the "program instructions" versus "input bits" meant, and I did not understand the notation of the finite state machines (especially figures 4c and 4d). I know it is always difficult to cater to broad audiences, but I think a sentence here or there to help would help, despite the word limits.

(Remarks on code availability)
The code base is well organized and I was able to run the code, although I did not extensively test it for errors.

Referee #5

(Remarks to the Author)
1. Summary of the key results:

The manuscript presents a DNA-based implementation of thermodynamically favored computation. Rather than driving computation through a fixed sequence of fuel-consuming kinetic steps, the authors design molecular systems in which the desired computational output is favored at equilibrium. The authors demonstrate that their scaffolded DNA computer (SDC) systems can compute accurately and rapidly across multiple programs. In the revised manuscript they have substantially scaled up the system, included new theory, new simulations, and a much clearer discussion of energy landscapes, kinetic traps, and the role of bind/replace pathways.

2. Originality and significance:

The theoretic idea of thermodynamic computing is not new, and the manuscript acknowledges this clearly. That said, I find the specific experimental realization presented here to be highly original. The novelty is not simply that computation can be mapped to a thermodynamic minimum, but that the authors have built a fast, reliable molecular implementation that scales substantially beyond a small proof of concept.

Having access to the first round of reviews, I can see that the authors have clearly gone to great lengths to improve the manuscript based on the reviewer feedback they received. They have directly addressed the questions about scalability, and they have modified the framing of their work to improve the contextualization. I find it quite compelling that the authors rose to meet the scalability challenge presented to them and implemented several 25-position (arguably more than 25 bit) algorithms! As a comparison, Woods' wonderful prior work published in Nature implemented 6-bit algorithms. Not only does the current manuscript represent a substantial increase in computational scale, but the drastic increase in speed also further strengthens the significance of the result.

The revised manuscript is also much better about not overclaiming. I do not read the current version as claiming that thermodynamic computation will replace silicon computers, quantum computers, or driven molecular computation more generally. Rather, the paper now makes a more defensible and more interesting claim: that molecular energy landscapes can be programmed and navigated to perform computation in a way that is experimentally practical and scalable beyond toy examples.

That conclusion is well supported, and in this revised form I think the work is appropriate for Nature.

3. Conclusions: robustness, validity, reliability

I have read most of the SI and I can't praise it enough. It is a fantastic example of "full-stack" nanotechnology development. Multiple levels of abstraction supporting a concept with beautiful theoretical work and leading it all the way to implementation.

I also find the comparison to DNA origami to be quite appropriate. Their approach has a similar practical appeal: simple strand-level design rules, good performance on useful timescales for reasonable systems, and relatively economical implementation (including the ability to use many unpurified strands without sacrificing yield). Of course, fully customized long synthetic scaffolds may become expensive for larger implementations, but the overall architecture feels experimentally accessible in much the same way that DNA origami was largely adopted because it "just works".

The authors are very clear that they don't enforce (or even rely) on particular kinetics. They hypothesize that many different kinetic paths leading to the same equilibrium state are what allow the system to compute so accurately and quickly. However, they are very clear that they mean to invoke discussion on this, and don't claim for it to be generally true (it just seems to work for their SDC schema).

4. Suggested improvements:

The most important revision I recommend is to complete the correction to the ADDITION experiments. Because the ADDITION experiments are among the central scale-up demonstrations in the revised manuscript, the authors should complete the corrected experiments and update the appropriate sections of the paper.

I concur with a previous reviewer that it is a shame that the very nice thermodynamic computing theory in the SI does not appear in the main text. However, I also think that the current version is well-suited for the general audience of Nature and the substantial rewrite required to incorporate those SI sections into the paper is likely not worth it.

One conceptual clarification that would be helpful is whether different output methods affect composability (whatever that may mean in a thermodynamic computing setting). For example, does using an output bit versus an output string change how readily the output of one computation can be used as the input to a subsequent computation? This does not affect my assessment of the current results, but it is related to scalability in some sense, and it may help readers understand how the architecture might be incorporated into larger computational workflows.

Minor errata:

Line 271: “We designed a …” appears to refer to multiple scale-up tests. Consider changing “a” to “some,” or otherwise revising the sentence for number agreement.

Line 304: “An M = 3 computation in is …” Remove “in.”

Lines 306–307: “covers over >97%” is redundant. Use either “covers over 97%” or “covers >97%.”

Figure 6 caption: “which prevents truncates error pathways” is unclear. Perhaps the intended phrase was “prevents/truncates error pathways,” or perhaps only one of those verbs was intended.

Acknowledgements: “We thank Ahmed Shalaby his fast …” appears to be missing “for.”

5. References: appropriate credit to previous work?

Overall, the background literature review is quite thorough, both in the main text and in the SI, and tells a lovely story of how this work fits into a broader effort to program molecular energy landscapes to compute. In particular, I appreciated the effort the authors put into S2 to connect the work to several neighboring literatures without making the background feel like a list of citations; instead, the discussion helps clarify what is genuinely new here and what longstanding ideas the manuscript builds on.

I do have one citation recommendation: Very recent work [1] seems to employ the thermal-cycling-reset concept together with the strand-displacement input-replacement technique to run new computations in a way that resembles the authors' renewable programs. However, [1]'s rechargeable circuits keep resetting back into a kinetic trap, rather than stable equilibrium. So, while the mechanisms and framing are distinct, [1] seems like a conceptual cousin of this work and would likely be a relevant addition to S8.1.

[1] Song, Tianqi and Qian, Lulu. "Heat-rechargeable computation in DNA logic circuits and neural networks." Nature 646.8084 (2025): 315-322.

(Remarks on code availability)

Open Access This Peer Review File is licensed under a Creative Commons Attribution 4.0 International License, which permits use, sharing, adaptation, distribution and reproduction in any medium or format, as long as you give appropriate credit to the original author(s) and the source, provide a link to the Creative Commons license, and indicate if changes were made.
In cases where reviewers are anonymous, credit should be given to 'Anonymous Referee' and the source.
The images or other third party material in this Peer Review File are included in the article's Creative Commons license, unless indicated otherwise in a credit line to the material. If material is not included in the article's Creative Commons license and your intended use is not permitted by statutory regulation or exceeds the permitted use, you will need to obtain permission directly from the copyright holder.
To view a copy of this license, visit https://creativecommons.org/licenses/by/4.0/

We thank the reviewers for the time and effort they put into our manuscript.

Summary of reply to reviewers:

- Scale-up up of system size from 4 to 25, included new general strategies for scale-up
- New Figure 6 with 3 scaled-up systems.
- New 0.6 pages of text on system scale-up
- Added context needed for scaleup in multiple places throughout the main text and SI, which includes development of theoretical model
- Modified and expanded Discussion
- New SI Sections: S9, S3.3, S10.2–4. Plus multiple modified SI sections (as detailed below)
- To make the text more suited to a general audience and to accommodate new material we removed technical details from the main text, expanded discussion of energy landscapes, and expanded prior literature. Reduced Figs 3, 4 and 5 in size.

-------- Referees' comments ------

----- Referee #1 (Remarks to the Author):

1. Summary of the key results
This article implements the idea of computing through thermodynamic annealing using DNA strands, demonstrating the concept through multiple steps of executing a finite state machine. they show that computing can occur accurately and quickly using this implementation for a range of computations and use these results to support the notion that computing by approaching thermodynamic equilibrium should be considered more broadly

2. Assessment of novelty

It should be said in fairness that everything, on some level has been done or thought of. It is also the case that in a short form article it is impossible to mention all related work.

Reply: Many thanks for the kind words. We thank the reviewer for their thorough reading of the manuscript.

There were a lot of individual items to reply to, in reading this, the reviewer may wish to first skip to the section on system scale-up (both experiment in Fig 6, and some theory touched on in main text and in SI Section S3.3), and then go through all other points, which are more focused on presentation of ideas, related work and conclusions.

With that said, there are two areas of concern.

First, the article makes it sound like all computers are iterative driven processes. It is certainly true that this is the dominant computing mode. However, the notion of thermodynamic computing is in itself not new. The idea of annealing to find an answer to a computation problem is the foundation of a family of statistical techniques, most directly simulated annealing. (This process is run on a computer but it is very much in the spirit here.) A general sentence or two (or section in the SI, though I hesitate to suggest so much expansion to the incredible body of work already there) of discussion about this field would improve the paper and the context. Further, in other areas of computing, such as quantum computing, I am not an expert but my understanding is that this notion of searching an exponentially large space to find a low energy configuration that represents an answer is a central concept there or could have central relevance in that searching through an exponential number of quantum states and preferring some due to, presumably, a lower energy state, is the fundamental advantage of quantum computing.

Reply: Thank you for the comment about related work. In the previously reviewed version we had referenced at least one paper on each of these topics, but admittedly we did not spend too many words on them. We tried to remedy things a bit, bearing in mind that a huge expansion of the paper happened due to other comments by the reviewer.

Action: Text has been updated in many places, along with better signage to the SI, e.g. the Abstract:

Like life, computers are out-of-equilibrium.[1,2] Thermodynamically favoured error states are thwarted by energetically-costly processes such as kinetic proofreading of biological polymers, error-correcting codes in computer data storage, and redundancy in molecular programming. Decades of theoretical work shows that unlike life thermodynamic computers can operate by drifting naturally to equilibrium.[3,4] Similar ideas underlie machine learning models[5] and search algorithms such as simulated annealing[6], although executed on non-equilibrium architectures at enormous energy cost.[7] Physically implementing thermodynamically favoured computation is a decades-long challenge that could reduce dependence on fuel-consuming error-correction and precise kinetic control. Here, we demonstrate a thermodynamically favoured Scaffolded DNA Computer (SDC) on ten programs including MULTIPLICATION-by-3, DIVISION-by-2, 8-bit PARITY-detection, and ADDITION of up to 25-bit numbers. SDC algorithms have simple experimental protocols, can be reused dozens of times and small instances run in under a minute. The SDC is grounded in mathematical, physical and computer science principles that explain why the output is thermodynamically favoured, why no error-correction nor precise step-by-step kinetic control are required and how it is progammable and scalable. This work creates a new way to think about equilibrium computation in all manner of synthetic systems. ·

The intro (has signpost to SI of the concept, along with the updated Fig 1):

Like living systems that consume fuel to stave off thermodynamic heat death, computers are typically out-of-equilibrium, consuming energy to enforce data integrity. The massive gap between theoretical energy lowerbounds[1,2,4,8], and what modern computing systems consume[9], leaves significant space to rethink computing infrastructure and algorithms[4,10,11]. To reduce this gap a perhaps counter-intuitive avenue is to design a programmable physical system whose equilibrium encodes the output of an algorithm, then allow the computer to simply drift to the right answer (Fig. 1, Supplementary Note S1). This notion has been presented in various forms[1,3,4,11] and stands in stark contrast to popular computing methods, so why is it not widely adopted?

The first challenge is finding a physical implementation that is computationally expressive yet suited to energy-landscape engineering and analysis. The second challenge is thermodynamic: the output state should be highly favoured, meaning the

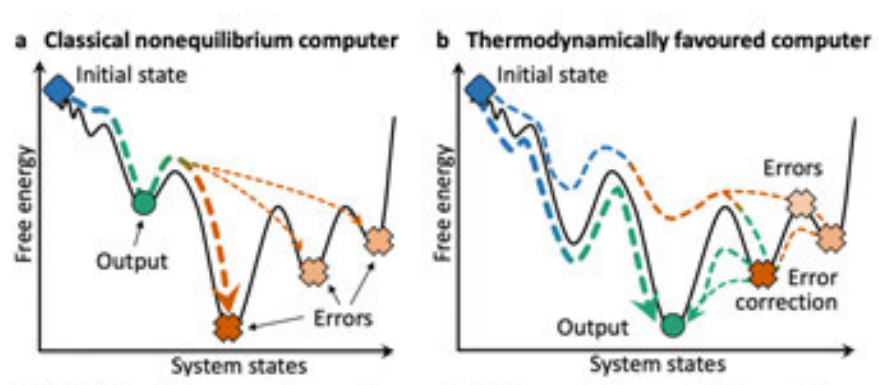

Fig. 1. Classical versus thermodynamically favoured computation. a. Classical molecular computers have an intended target/final state that is out-of-equilibrium. Errors show up as unintended nucleation of incorrect structures in algorithmic self-assembly[13] or leak in strand displacement circuits[16]. Digital electronic computers have similar issues, where decades of research have lowered error rates but with large energetic costs[9]. **b.** Thermodynamically favoured computation has the output state being the energetically most favoured. Hence, errors are automatically repaired by the system naturally going to equilibrium, although avoiding kinetic traps is a challenge (Supplementary Notes S1, S2).

Suppl Note S1 has been updated, as follows:

Algorithms inspired by thermodynamic energy-based principles The long and intertwined history between computer science and physics is exemplified by the large number of algorithmic ideas inspired by thermodynamics and ubiquitous energy-based models [34]. A simple example with a decades-long history is simulated annealing [35] takes a straightforward approach, where candidate solutions to a search problem are assigned an energy, with better solutions having lower energy. A randomised algorithm navigates the solution landscape, with a time-varying temperature parameter controlling stochasticity: typically starting at high temperature to widely traverse the search space while avoiding getting trapped in local minima, then lowering the temperature to optimise within an minimum. As with *all* algorithmic ideas, success is a function of the match between algorithm and problem, with computational complexity theory being the ultimate arbtrar on what is efficiently solvable [33]. That is just one example, there has been a huge amount of research on Energy based model, see for example [34], with the cannon including simple models like Hopfield neural networks and Boltzmann machines, as well as more complex deep-learning and generative models.

5

There is also an inspiring history of using computer science ideas to show hardness of problems in physics [36], by showing that those problems, whether we like it or not, embed algorithms. Since computation represents some of the hardest, yet reasonable, dynamics we can imagine, this endows those physics problems with unpredictability [33].

We could expand this further of course. But hopefully the pointer to a decent survey should work. Suppl Note SI.2 also contains other relevant related work, not shown here.

Finally, quantum annealing is referenced in the Discussion (only one word, sentence -2), but we think more importantly the Discussion is now giving the broader picture and context for the work that the reviewer desired (see comments below).

Second, I think a couple of fundamental points were missed with respect to other methods of DNA computing and there are a couple of missing references about similar work. Two stand out:
a. The authors state that algorithmic self-assembly is not a thermodynamic means of computation because it requires nucleation. However, that requirement for nucleation is more correctly understood as a means to efficiently compute the thermodynamic minimum by providing a kinetic pathway toward that minimum. Conceptually, this reviewer is not convinced that at equilibrium there would not be the same preference to a perfect computation (because of the need to minimize mismatches) in this process. This process would in fact directly map to Woods' beautiful recent work in Nature on algorithmic self-assembly (2019). In this, I find the claims about algorithmic self-assembly misleading.

Reply: We essentially agree with the reviewers argument for the related case of non-algorithmic tile self-assembly, but for the case of algorithmic self-assembly we respectfully disagree. The issue is due to an additional complexity only found in algorithmic tile sets, and perhaps specifically in how they have been experimentally implemented to date: extra unused tiles floating in solution that should **never** form an assembly, are themselves out of equilibrium. This is an admittedly unique and bespoke issue, and we spent quite some time figuring out how to highlight it more clearly.

Also, for clarity of discussion: We believe the reviewer is talking about SI Section S2.2.1 and not the main text, since the main text completely avoided details about how spurious nucleation arises in algorithmic self-assembly. In the previous version, S2.2.1 gave 3 reasons why algorithmic self-assembly is out of equilibrium and/or has issues relative to SDC.

We should add that for those tile types that should be in an assembly, they can be said to go to equilibrium (setting aside some caveats about equilibrium error rates, and pathways to resolve those errors).

Action: To more clearly address the reviewer's comment we have now completely rearranged SI section S2.2.1, making the out-of-equilibrium point more clearly on only **one titled paragraph**. We hope this is more clear now!

Based on the reviewer's comment we have rewritten Section S2.2.1 completely. We added paragraph headings to separate out the concepts and improve clarity:
1. The argument about non-equilibrium behaviour in algorithmic self-assembly is hopefully clearer now: we changed the text so that the argument is confined to more clearly be only about how *excess tiles* behave in the two systems—something we might call the *primary* driver of non-equilibrium behaviour in algorithmic self-assembly. The paragraph headings and structure should have also improved clarity of the section, changes in blue:

> **Spurious nucleation** Spurious nucleation of off-scaffold structures is a fascinating major challenge and open research theme for tile-based algorithmic self-assembly.[13,81,94,95] A technical issue that is specific to algorithmic self-assembly, as implemented to date, is that after a correct computation has grown—from the seed all the way to completing its computation—there are necessarily large excesses of tiles in solution, which are available for the error mode of spurious nucleation. In correct computations, these tiles are of a special type that should not be bound into any structure whatsoever, they must be left free-floating otherwise the system is in an error state. For example, running a bit-copying nanotube from ref.[13] on input bitstring 000000 would mean that the intended final system state has (i) collection of seeds copying the string 000000 ("the correct answer"), and (ii) a huge number of excess free-floating 1-tiles in solution that should not be bound to anything ("the unused tiles, only useful for other inputs") Unfortunately, those 1-tiles can be considered an out-of-equilibrium system by themselves, sitting, waiting to eventually spuriously nucleate, at the growth temperature (because the system is being held in growth-favourable conditions, temperature, tile concentration, etc.). This kind of error mode is avoided by the SDC, since (a) SDC compute tiles only bind weakly to each other (hence after all scaffolds are coated with tiles there is nothing for them to bind strongly to) and (b) we would never drop to a temperature where such compute strands aggregate off-scaffold.[3] In fact, we can be much more liberal with temperature in the SDC, as the next paragraph explains.

2. A point about fine-tuning/sensitivity comparisons has been expanded a bit, and is under its own separate heading now, so that it is not confounded with the out-of-equilibrium argument.
3. We completely removed 30 lines of text that went into a subtle argument about potential differences in logical error rates in the two systems. This probably demands a full theory paper which could take years. Thermodynamics of algorithmic errors is super-interesting, we'll leave it to a later time.
4. Finally, we expanded the text on both intellectual inspiration and challenges we learned about through working on algorithmic self-assembly, several of which inspired our work here, to try to make it abundantly clear we are indeed very much inspired by that work. (We were concerned the reviewer thought we were "dissing" it, Two co-authors have devoted significant parts of our

lives to it so we're perhaps *too familiar* with the technical challenges of this thing we love, so the text has been recalibrated.)

b. There are other recent articles about using thermodynamic minimums to compute logic functions. Most notable is Nikitin, Nature Chemistry, 2023. This article also has weaknesses but the authors need to address distinctions in their approach, though it is recognized that the comprehensive treatment of the subject here is interesting. This article might also provide encouraging points about scaling of the processes studied here, which are a critical challenge about significance (described below).

Reply. The reviewer is correct: we have tried to do much better on this, and have modified the text. See Action below. [For context, the previous version did have a full sentence on Nikitin's paper in the main text: "Shallow equilibrium Boolean circuits were demonstrated in DNA using weak molecular affinities, but the challenge of fuzzy equilibria for scaling to deeper circuits remains\cite{nikitin2023}.", another citation to Nikitin in the Discussion, and we dedicated all of SI Section S2.3.2 (which was 21 lines of SI text), to Nikitin's paper.]

We agree Nikitin's work is very interesting, yet has limitations. One could do a full theory paper on his model, we conjecture that if done in a different way the approach could be significantly strengthened. First, by modifying the design to make it better suited to energetic prediction by the nearest-neighbour model via nupack/viennaRNA (Nikitin struggled with the weaknesses of those models for non-duplexes, and ended up trying random sequences in the lab rather than via sequence design, which in turn we think contributed to weak separation of 0/1 signals). Second, more speculatively, some sort of correctness-amplification gadget between the layers could be used to lower errors and thus improve the separation between 0 and 1 signals. There are likely many other ideas. A lot of conjecture and potential future work.

What we were trying to politely hint at with our admittedly vague terminology "the challenge of fuzzy equilibria" is the fact that Nikitin's shallow circuits of only depth 1,2 & 3 show 0 and 1 signals being close to each other (poor yield/signal-to-noise). (His 500 "bitness" gadget is in simulation only, so we're not including that here.) Quantitatively, the outputs of these shallow circuits seem significantly worse results than similar depth SDC computations e.g. depth 1 (single gate) of Nikitin's circuits) seem to look worse than depth 1 to 4 of SDC circuits. This shows up in Nikitin's square root result—in many cases, bits 0 and 1 are very close to each other in terms of output signal, e.g. sqrt(7) versus sqrt(9), for only a depth 2 or 3 circuit, with 4 gates.

To be clear: we think a proper comparison with Nikitin would itself be a full paper. And maybe his system should get more attention on scaleup for a better comparison, e.g. put in as much effort as we did here. Expanding Nikitin's system up to the level of programmability and size-scale-up and speed as ours (depth 25) seems extremely challenging, but we really don't know. We took the following actions:

Action: We shortened the introduction sentence to read: “Shallow equilibrium Boolean circuits were demonstrated in DNA using weak molecular affinities\cite{nikitin2023}.” but expanded the topic by adding two sentences to the conclusion:

time, dynamics while driving to a ground state—a fixed-count chemical oscillator[19] being an example. Equilibrium computing by strand commutation[26] encodes successive Boolean circuit layers as a cascade of weaker binding, base-mismatching, strands. The energy landscape is challenging to control due to DNA binding model uncertainties, but theory may show how to amplify the signal.[3,55] Beyond molecular programming, the theory of thermodynamics of computation[1–4,8]

Finally, we improved/expanded the supplementary text in Section S2.3.2.

S2.3.2 Strand commutation using non-complementary binding networks

Nikitin [55] recently proposed a system called “strand commutation’ that uses partially-complementary strands: the idea is to have a set of strands that weakly bind to each other, and let that binding networking go to equilibrium. Introduction of an input strand, that binds strongly to many other strands, will switch that equilibrium, which can be exploited to build logic gates. The system is novel, expressive and quite fast. Like the SDC system, the use of thermodynamics as the main driving force, and an apparent lack of unintended kinetic traps, leads to rapid processing of information.

Although both strand commutation and the SDC utilise the idea of letting the system go to equilibrium, they have a number of key differences, in particular information-encoding is quite distinct between them, and the two systems program different kinds of equilibria: for SDC the system output/readout is designed to only picks up scaffolded structures, and we have levers (compute domain binding strength, tile concentration excess) to drive, or program the intended/correct structure to be extremely favoured. In the strand commutation system there is a more ‘mixed’ equilibrium where intended and unintended results may appear together in a mixed population but with the intended winning the vote. With strand commutation there could be room to design clever gadgets to suppress error, between circuit layers, possibly taking inspiration from kinetic proofreading for algorithmic self-assembly [23], or leveraging ideas from the Thermodynamic Binding Network model [91].

Also, as noted in [55], thermodynamic DNA sequence prediction algorithms, and their parameter sets, are likely more accurate for perfect duplexes than for partially complementary strands, or even for systems that use fully complementary partial-strand domains with nicks, overhangs, geometrically constrained loops, etc. There seems to be scope to improve that situation, with clever design/abstraction improvements such as those by Berleant [95, 96], or perhaps by using modern high-throughput laboratory techniques to find better DNA sequence parameter estimates. We should add the major caveat that both strand commutation, and SDC, can be modified/extended in so many ways that future systems could crossover to utilise features of both simultaneously. There remains space for a full theoretical characterisation of the abilities and limitations of the approach.

3. Assessment of significance

This paper is notable in its enthusiasm and original discussion. It stimulates thought and forces one, as great science should, to think big picture and perhaps how this field (unconventional computation) might make large strides.

Reply: Thank you for the kind words.

That said, I think that the paper's ultimate contribution is less dramatic than it might first appear. This issue is not, specifically, the challenge of novelty per se, though the issues described above are important to address.

The issue is rather the question of the ultimate benefits and power of thermodynamic computing, which do not at all deserve the treatment they deserve in such an article.

Figure 1 is a great visual and it does capture the magic of thermodynamic computing, which is nice. But it also misleads the reader, as the figure makes it seem as if there is only one error state. This is correct if computing were only one step. However, in a computing processes that is, whether iterative or a search that requires either computing space or computing steps (one or the other must grow as the complexity of a computation increases, speaking in the roughest terms), the size of this space increases exponentially and a computation must decide between one of an exponential number of trajectories.

Reply: We've taken a lot of actions to this point and related points below.

Action: We have modified Figure 1, so that there are several error states now, and explicit mention of kinetic traps in the caption.

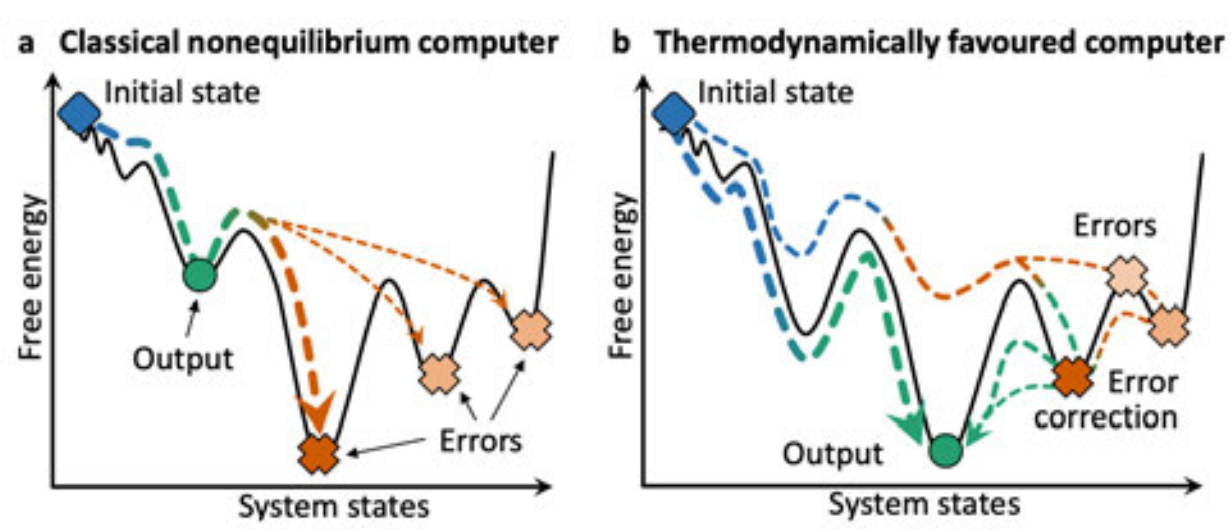

Fig. 1. **Classical versus thermodynamically favoured computation. a.** Classical molecular computers have an intended target/final state that is out-of-equilibrium. Errors show up as unintended nucleation of incorrect structures in algorithmic self-assembly[13] or leak in strand displacement circuits[16]. Digital electronic computers have similar issues, where decades of research have lowered error rates but with large energetic costs[9]. **b.** Thermodynamically favoured computation has the output state being the energetically most favoured. Hence, errors are automatically repaired by the system naturally going to equilibrium, although avoiding kinetic traps is a challenge (Supplementary Notes S1, S2).

And we've rewritten the Discussion to more clearly talk about the energy landscape, and potential pitfalls. The new figure 6 has a state-space energy diagrams showing potential problems and how one of our systems overcomes them. There is about 0.6 of a page of new text, for system scaleup, that is all couched in the language of energy landscapes and needing good kinetics through those landscapes. Rather than pasting here, we felt it easier for the reviewer to read directly there with proper context.

In "driven" computing, this decision is made by a ratcheting effect that each of a set of steps is irreversible. As such each step must be decided cleanly (at the cost of energy) but the upshot is a benefit in terms of time taken to compute - it scales linearly with the number of computing steps even as the number of potential trajectories increases exponentially. The authors are correct that this costs energy.

In thermodynamic computing as the authors describe it, this exponential number of potential trajectories must all be considered at once. The energy minimum can be found, but the challenge of doing so increases exponentially with the number of computing steps in the finite state machine.

Reply. Point taken. The reviewer is 100% correct that if our system had some kind of exponential large flat-ish landscape, we'd get nowhere, instead the system would wander around for time exponential in scaffold length. Even worse, any implementation would not even be flat: the landscape would have unintended bumps and dips, compounding the situation. Thankfully this is not the case for our SDC model, nor, it seems, our experimental implementation.

Action. We have made significant additions to the paper to demonstrate both the experimental and theoretical plausibility of efficient navigation of the exponentially large energy landscape in the SDC model. Theory: These changes include a new SI Section with a Lemma (Lemma S3.2 in the SI) and computer simulations (Fig S1) to show that there are simple models, consistent with the bind/replace kinetics suggested in main text Figure 2, that navigate the exponential large state space efficiently, in time merely quadratic in scaffold length. Experiment: described below, but we've done significant scale-up work. These updates are also in 3 places in the main text, plus figure 6 of main text.

As an aside, it is interesting to note that random walks have a long history in the thermodynamics of computing–being one of the tricks used in Bennett's [1973] beautiful reversible simulation of non-reversible Turing Machines.

This challenge has been considered, for example in Levinthal's paradox in protein folding. In Levinthal's paradox, however, the solution is actually to return to the kinetic perspective and to observe that energy landscapes without a clear "folding funnel" are selected again, though my understanding of this area is limited. Such a "folding funnel" would, however, be exactly the kind of "pathway engineering" the authors seek to avoid here. This suggests that the problem with this idea is in its scaling -- it works beautifully with 5 steps, where there are perhaps on order 3^5 = 243 configurations, but once one goes to even 10 or 15 steps the number of trajectories that needs to be distinguished is untenable - rapidly approaching millions or billions as the complexity of the annealing process increases exponentially.

The fundamental limitation to this paper is the authors' systematic ignoring of this key fact to at least temper their discussion, which does a disservice to the field. Undoubtedly. the results are neat -- the implementation is elegant, and the experiments and results are clear and well done.

Reply. We have changed this emphasis of the article completely now. As noted in previous replies above. As the reviewer says, our solution was to "to return to the kinetic perspective". We continue:

But the emphasis of the article confused me. The key question I had was "what if you did 10 or 20 computing steps"? Does this really work? This is not that hard to do -- modern synthesis could easily produce the scaffold needed for each of these processes, and the longer strand would be the only step needed. I would fine 2 demonstrations of 10 or 20 step computing process more compelling than the panel in Figure 5 which honestly prove the same points as the results in previous figures are not that surprising. Having a longer scaffold would also allow a proper study of the time needed rather than an extended discussion of maxing out the speed of the thermocycler used. The experimenters were clever but right now what their experiment suggests is clearly "less than 20 minutes" for 5 steps for a great answer and a mediocre answer in just 1-2 (which is exciting, to be clear!) but could easily be studied by looking at different 8, 10 or 12 etc step processes and different annealing speeds. If, indeed, the scaling is surprisingly great and trackable (see next paragraph), that could be something to report in Nature.

Reply: We admit that we groaned a little when reading this. :) But we accepted the challenge. And now our paper is significantly better for it. Thank you. We are immensely proud of what has been achieved, we hope the reviewer likes it!

Action: The quick summary is that we scaled the system up to length 25, five more more than the reviewer asked for. There is significant new text in the paper, a new Figure (Fig. 6), and a new SI sections (Section S9 has experiments, and Section S3.3 has theory, S10 has ~1.5k new DNA sequences), The main text also has sentences in several places talking about bind/replace kinetics, as well as changes to the Discussion.

To challenge the system we used cheaply available M13 as a scaffold; we put significant effort into choosing the best segment of M13 to use, using a thermodynamics-based sequence design toolkit we developed, but compared to other computing systems' sequences M13 scaffold domains are energetically terrible (in algorithmic self-assembly, and for SDC compute domains here, we try to design domain sequences/strands to be isoenergetic within a window of 1 kcal/mol (usually way less, usually within like 0.2 kcal/mol which is beyond the accuracy of the NN model), but the best we could get with M13 was about 10 kcal/mol (NUPACK MFE, duplex binding, at 65 C). Also, to save on material, we ran the system at 10x less concentration than before. And of course with M13 there was ~6.6k of single stranded DNA just "hanging out" for all of our computations. Despite those 3 non-idealities, the system seems to have worked pretty well. Oh, and we fired ahead with all reporters and compute strands unpurified…should work, right? What could possibly go wrong?

Figure 6 has three new panels. Fig 6b starts with BITCOPY on scaffold length 11, and shows one scaleup strategy (scaffold base mismatches to improve M13 energetics), that gave some, albeit limited improvement. The part we are most excited about are where we developed (pretty simple) programming-style techniques to combat non-isoenergetics in the system. The ADDITION program, in its logic, has a natural error suppression characteristic. Our ADDITION computations add two 25-bit numbers in a handful of hours (50 bits of input!). [redacted]

[redacted]

[redacted] For programs that don't have that logical structure, we came up with a rather simple and very general domain-shift, or domain-renaming, trick that balances energies in competing configurations, exemplified in the ISOENERGETIC-BIT-COPY in Fig 6 (and SI Section S9). Here is an example from Fig 6:

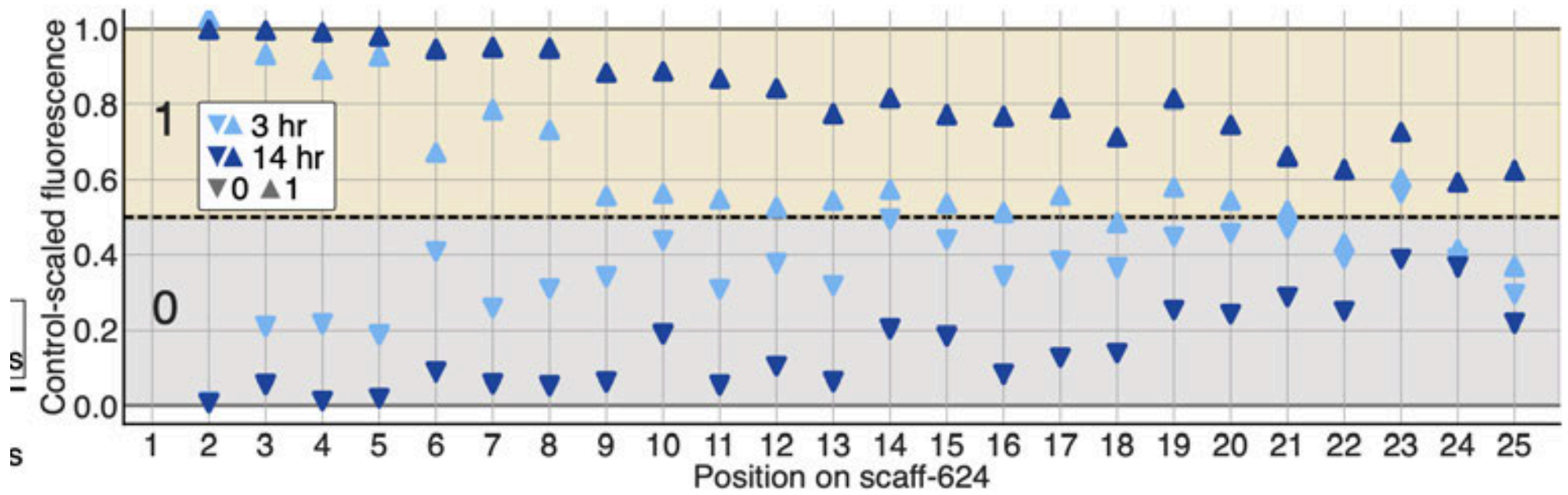

We have not yet encountered obvious barriers to further scaleup. Hence, we believe there is room for more to come.

Two minor points:

1. We changed how the fast data is presented, by moving it from Fig 3 to 6. So now "speeding up" and "scaling up" are together in Fig 6. Previously the fast data showed 4 ADDITIONs, it now shows 4 different programs, all running in a minute, which gives a better sense of how speed affects different programs.

2. We carried out over 650 new experiments, plus controls, using a custom developed code pipeline, with an Echo liquid handler and qPCR machines which provide very efficient way to work. However, there is a small mistake (human error) which we will correct in the next version of the paper: position P in most ADDITION experiments is missing data due to a copy-paste coding error. We'll repeat this experiment soon.

One could also argue that the number of steps is not the point -- of course it will stop working at some point. But to that I would say, first, that it is the point -- if it works well for a 50-step computation, that is vastly more exciting than working only for 5 but not for 8. If the former, it suggests that most computations we do with molecules now or wish to do for a range of biological processes, could be done easily by annealing and complex chemistries maybe aren't needed. Second, the number of steps being finite demands discussion as in now universe is this number sufficient to for example consider annealing to replace silicon or quantum computers for difficult calculations. With this in mind, how might thermodynamic computing here be visualized as a component within a bigger process? This should be addressed with at least a sentence or two in the impact section beyond "This is a conversation piece" if this is really an article for general interest, as the authors have more expertise than the general reader on what the impact is here.

Reply. Interesting points. Hopefully these points have been addressed in the main with our scaled-up results. We also used the reviewer's comments to try to take a more general

approach in the discussion, focusing in a few places on the take-home message from our scale-up achievement. I.e. programming the energy landscape. One example being the "stepped" stepped energetic landscape that Isoenergetic-Bit-Copy defines, another is the energy landscape navigation that ADDITION provides. We hope that these ideas bring new ideas to the conversation about thermodynamic computing, with molecules of course but also to other technologies like quantum computing.

Some modifications to the Discussion are follow:

data reported in Fig. 6a, MULTIPLYBY3 and PARITY.

Intuition suggests equilibrium computation could be imprecise, since any desired output configuration competes with exponentially many off-target structures. We outline thermodynamic and kinetic arguments against this intuition for the SDC. Our thermodynamic design principles engineer the predicted target (MFE) structure to have probability approaching 1.0, driving the sum of probabilities of all other structures to 0 (Fig. 2f, Supplementary Note S4.3.1). Indeed, the correct SDC output was reported in all experiments. Notably, average completion/yield on typical-anneals, at scaffold length ≤ 4, was 95.3 % (SD=0.035) of strict controls with fewer strands and simpler equilibria. Mean yield at scaffold positions B and C was 98.1 % (SD=0.014), dropping to 94.4 % (SD=0.034) at D. Increasing competitive complexity from 2 to 8 did not significantly degrade yield for typical 3-hour anneals.

For larger N, mere thermodynamic favourability may not be sufficient: we need a nicely-shaped and traversable energy landscape. The abstract SDC tile model gives a stepped landscape, efficiently explorable by random walks that ratchet forward via compute domain mismatch repair. Hence, in theory and practice, we took a somewhat liberal approach to kinetics. The bind/replace model in Fig. 2b and Supplementary Note S3.3 begins a computation by guessing one of a large set of $2^{O(N)}$ initial states, yet by mere random walk completes in only $O(N^2)$ time. However, experimentally, we did not enforce this particular kinetics, nor any other: we simply annealed the system, hypothesising that something akin to bind/replace is happening at higher temperatures. We used a strand design without intentional kinetic traps (Fig. 2), but did not carry out design optimisations, nor did we seek to characterise and prevent off-pathway interac-

computations[26,43–45]. Results for our super-fast, and typical, anneals suggest the SDC has fast, high-temperature, reversible assembly kinetics (Supplementary Note S4.3.2). Scaling-up to to scaffold length 25 systems required slower anneals. The awkward energetics of cheap biologically-derived scaffold (M13) slowed computation at some positions, but in a way that seems not unlike our proposed random-walk kinetics (Supplementary Note S3.3). We conclude that the SDC is robust by designdespite much remaining to be understood.

Although the earliest DNA motor was reusable[46], designing

…

proof-of-principle for under-designed DNA sequences.

Our work shows that DNA systems are amenable to free-energy landscape programming enabling equilibrium molecular computing to move from theory to practice. But more theory[1,3,4] is needed to understand the advantages and limitations. Fuel-consuming, infinite-time, out-of-equilibrium molecular dynamics[1,19,46] are, by definition, unsuited to ther-

4. Other comments / improvements

Line 3 of abstract: "Unlike life, decades of theory shows" -> "Decades of theoretical work shows that unlike life," **Action**: Done.
Line 4 of abstract: "can execute" without a direct object is awkward **Action**: execute -> operate
Line 7: remove comma after "a" **Action**: Done.
Line 42: What is fuzzy equilibria **Action**: We have removed the wording altogether (two places in the introduction).

Figure 2f : From reading the SI the x axis caption should be "temperature" and the idea is that this is the probability of being in the right configuration given that the system reached equilibrium for that temperature. The caption should be improved to clarify what is being plotted.

Action: Changed Figure and caption as requested.

Also, SI Section S4.3.1 has more details on how the plot was produced (a custom partition function algorithm needed to be developed, as dynamic programming algorithms like those implemented by NUPACK/ViennaRNA do not scale well for multiple strands, since multi-stranded MFE is NP-hard [Condon, Hajiaghayi,Thachuk; DNA27, 2021]).

The authors allude to origami as a means of thermodynamic annealing that scales to a large number of strands. It is true that large DNA origami can fold well but there are several key points about that the authors leave out which really diminish the power of this metaphor to believing that this process will scale well:
1. Origami folding is highly cooperative (Dunn et al, Nature, 2015). This fact makes origami folding more efficient than the assembly here as the number of strands increases. But it also points out that origami folding explores far fewer potential configurations than this process aims to and so (as that article shows) will not necessarily find the thermodynamic minimum in a short time period.

Reply: Although the 1D SDC studied here is not cooperative in the same way as 2D/3D origami, we hope we have demonstrated in the updated text, Figure 6, and SI, that the SDC can be designed to have both nice energy landscapes (e.g.) and to have efficient pathways through that landscape.

1. The pure/abstract SDC tile model has at least one simple kinetic model (bind/replace) runs in O(N^2) time for scaffold length N (for, say BITCOPY).
2. Non-idealities in scaffold domain strength (because we used M13) and unintended differences in compute domain strength, could thwart that, but we designed Isoenergetic-Bit-Copy to illustrate just one way to programmatically optimise the landscape.
3. The ADDITION program illustrates how the logic of some programs should give even faster kinetics than BITCOPY. Even if one does not believe the system uses bind/replace kinetics, it seems likely that many reasonable kinetic models wold predict ADDITION is much faster than BITCOPY.

As an aside, our future design ideas for 2D will exploit the beautiful cooperativity that Dunn et al investigate.

Action: The paper is now more centered on programming the energy landscape, and then navigating it. Fig 6 and the associated page of new main text creates a story along this theme. The Discussion comes back to this topic in three places, as already noted.

2. Even despite the limited space of exploration, the long folding times needed for naively designed DNA origami (cf Douglas et al, Nature, 2009) can be extreme -- weeks, suggesting also that scaling here will not be favorable.

Reply: We hope the reviewer agrees that these scaling points have now been addressed – see earlier replies, along with our experiments (and preliminary theoretical hints). We ran 25-position experiments in 14 hours (Iso-Bit-Copy) and 24 hours (ADD, for more than 99% of "input hardness" as measured by *M*-value), with a really quire suboptimal scaffold (M13). We're already getting ready to work on synthetic scaffolds, custom designed, and maybe then we'll aim for even larger scale-up, and 2D of course, but we're good for now I hope!

There is a bunch of what I might term "jargon" in this paper, such as CG clamps, and it doesn't generally feel written for the general reader. For these and other reasons, most importantly maximize the impact of this work, I suggest a journal such as Nature Physics rather than Nature for this article (see next section)

Reply: We tried to significantly lower the amount of jargon in the paper. There are probably too many to list here.

Action: We won't list all, but certainly more than 10 places we removed technical terms and/or reduced jargon, including rewording the GC clamps sentence to "DNA sequence tricks", removing the 3 technical-sounding jargon (mismatches, GC-ends, clamps), and removing quite a bit from the Discussion and reducing technical field-specific terms in the introduction. Given the massive expansion of the work, it was helpful to have this guidance as to what we could reasonably chop. Despite all our efforts we are likely still well over the typical word limit, any further guidance to ensure the general audience finds points of interest are of course appreciated.

5. Appropriateness for this journal

This paper is exciting and refreshing in its genuine enthusiasm. Reading it over, I felt that some of its weaknesses with respect to publication in Nature might be strengths for an alternative journal choice, which would be Nature Physics.

This recommendation is in part because this paper does not represent the jump in the capability of the model over prior work -- thermodynamic computing has been done in less elegant forms before and the demonstrations here do not provide a surprising increase in the capability of the process over prior work.

Reply. With the scaled-up programs, we hope the reviewer might be more convinced now. By the metrics (A) of number of steps (or circuit depth), 25, (B) or number of input bits, 50, we believe nothing comes close in the field. Likewise (C) for number of repetitions (ran 25 times) on the renewable work. Comparisons with some prior work are explained in the Discussion. We did

not think it constructive however to compare very different computing paradigms by these metrics in a quantitative way, since SDC is a new way of computing there is no “in model” comparison to do, and the other models (strand displacement circuits, algorithmic self-assembly, computing on DNA origami surfaces, etc.) are quite conceptually different in goals and who they do things. This could be reconsidered however.

We do believe our system is significantly more computationally powerful than any of the (very few) thermodynamically-favoured computing gadgets out there, and non-term favoured also. Implementing a 25-bit adder would be a serious step forward in TMSD, algorithmic self-assembly, or other molecular computers we can think of.

It is also because, fundamentally, I disagree with the claims made in the abstract that this article will lead to reduced energy consumption. The authors' results do not support that. Thermodynamic computing costs energy because one must wait for the computation to complete and maintain specific environmental conditions to allow that to happen. The exponential increase in this time is likely to make this result of more academic interest than of any immediate practical interest. Unfortunately, the article, by skirting this fact rather than facing this real question and trying to contextualize the results rather than making a lot of a small demo, could end up shutting the door on this direction before it should be shut (and which happened, perhaps, as a result of Adleman's seminar 1994 work, which I was disappointed not to see cited here).

Reply: We largely agree with the reviewer. It was not our intention to claim that the system will lead to reduced energy consumption overall, rather that the specific kinds of error-correction systems that we’ve seen developed over the last 2 decades in the field (which have energy and scale-up costs) may not be needed. There will be a trade-off, our looser random-walk kinetics means that there is less energy-consuming step-by-step ratcheting, but the kinetics is still guided by a nice landscape, which has a cost. We’ve not needed a “proofreading” or “leakless” style scaleup gadget thus far, for example, there are some energetic distinctions to be made between SDC and other DNA computers. We are simply trying to open up the conversation of energy consumption in computing, and molecular computing in particular. These kinds of ideas could in fact be applied in silicon, or who knows what else.

Actions:

1. Abstract: changed **“**obviate fuel-consuming error-correction” to “reduce dependance …”.
2. Abstract: Changed: “By demonstrating the concept of thermodynamically favoured computation, this work creates a new way to think about energy-efficient equilibrium computation in all manner of synthetic systems.” To: “This work creates a new way to think about equilibrium computation in all manner of synthetic systems.”
3. Also Fig 1 is very prominent/close to the abstract, and has been changed to add in some of the energetic complications (multiple error states are now shown, and the caption adding “although avoiding kinetic traps is a challenge”).
4. Finally, the Discussion has been largely rewritten, as mentioned before.

However we felt it best to leave the wording of the penultimate sentence, as a stimulation for discussion and a challenge for the field and other fields, including quantum and (more) classical digital-electronic “thermodynamic computing”.

We added a citation to Adleman’s paper as requested (although we are over the permitted number of references.)

But I also think that appropriate placement of this paper will increase its impact over placement in Nature, where I feel it will create confusion and perhaps even backlash, neither of which is needed. The authors state, and I agree mightily, that this paper is a great piece for discussion for advancing the world of nontraditional computing. And that the need to think about new approaches to unconventional computing is also great due, yes, to the extreme challenges of energy costs.

Its SI ,which in itself has a lovely review of the field (and really should appear as a separate piece, honestly) is great context. The careful analysis of the thermodynamics should be in the article itself, as it is much more important (in my opinion) than the various demos (after 2-3) in Figure 5 in terms of advancing the work -- it is a coherent theory that is lovely and really does provide a place to move this field forward and think about how to build better molecular (and other) computers in a systematic yet creative and free way. But the way this article is structured now, this contribution is not at the center as it should be.

Reply: Thank you very much for the kinds words about our SI. We enjoyed the phrase “think about how to build better molecular (and other) computers in a systematic yet creative and free way” – thermodynamic-favoured computing can indeed be rather liberating in ways. The comment “this contribution is not at the center as it should be“ might involve a possible major rewrite by moving SI material to the main text and thus necessitating removal of large chunks of main text (due to word count limitations), we would humbly ask that the reviewer first asses all our other changes and expansion to the article, and if their comments have been otherwise adequately addressed. If they then still feel strongly about this point we will do our utmost to address it. We hope this sounds reasonable. The reviewer is making us ponder if there is room for a review article in the coming years.

Referee #1 (Remarks on code availability):

I read some of the SI but again, in the interest of providing feedback on the significance, did not read all of us but could do so, though I would want to ask a postdoc for help to do a good job.

Many thanks for the thorough and thoughtful review.

----- Referee #2 (Remarks to the Author):

In this paper the authors put forward a potentially new (and general) strategy for error free computing. This strategy is based on using the thermodynamically preferred ground state as a basis for computation. A contrast is drawn to the so called typical paradigm in which the end result of the computation is not the thermodynamic ground state but rather a metastable state.

There a lot of interesting points in this paper but I find a few key points confusing. The entire paper is seemed to be based on the premise that a large class of computation outputs can be mapped onto thermodynamic ground states. Is this always true ?

Reply: Yes.
There are a few reasons, in differing domains:

1. Classically, one of our main inspirations is Bennett's work on showing that reversible Turing machines are universal. He used a small constant bias (or energy) on the rates to give give constant probability of the correct output (our recent unpublished theoretical work replaces that bias with a mere 3x increase in state space size to get the same result (https://arxiv.org/abs/2309.06957).
2. Energy models in machine learning. This is a bit more nebulous, with a huge amount of literature. For nicely shaped energy landscapes, stochastic algorithms can navigate them (for the SDC we get to design the landscape, not always the case for stochastic algorithms where you are handed some hard problem to solve, e.g. NP-hard optimisation problems would necessarily have bad landscapes, assuming P =/=NP).
3. More close to home (for us) theorists have been thinking about thermodynamically favoured computing for a while now, e.g. Doty, Rogers, Soloveichik, Thachuk, Woods. *Thermodynamic Binding Networks.* DNA27. https://arxiv.org/abs/1709.07922

That is merely to make the point to the reviewer that there is nothing contradictory or strange about the nation of having the output of the computation be the result of a system going to equilibrium. Although it certainly has strange and interesting consequences when compared with existing forms of computing.

Doesn't frustration due to mutiple competing pathways make computing with metastable states a possibility always ? In saying this, I am considering computation done in/using/by the Hopfield inspired models.

Reply: Avoiding metastable states is a key concern. A good self-assembly example to consider is DNA origami: a remarkably successful design/system where the ground state is favoured, and typical (2D) designs do not get caught in erroneous/metastable states, The SDC takes direct inspiration from DNA origami, exploiting some of the same ideas (scaffolded assembly, excess concentration of small strands), and bringing new ideas (encoding information and logic in compute domains). We design the SDC landscape to avoid metastable states.

Action: In the updated version of the article, in many places, we talk about the energy landscape of the SDC system and how an executing SDC program moves through that landscape. Examples include:

temperature-dependent, pathways.

Under bind/replace kinetics the expected computation time is upper-bounded by $O(N^2)$, the time for the leftmost compute domain mismatch (error) to disappear by a random walk to scaffold position N assuming a single anchor tile (Supplementary Note S3.3). However, although we defined one reasonable kinetic model, we intentionally do not experimentally enforce any particular kinetics, a *laissez-faire* approach that stands in contrast to DNA computing using toehold-mediated strand displacement where precise domain-level or even base-leve kinetic pathways are engineered.[16,30] We hypothesised that the combination of thermodynamic favourability and multiple plausible kinetic pathways would enable a straightforward temperature anneal to overcome kinetic traps, unlike previous carefully optimised systems that use temperature holds.[13–16,18,20,21,23,24,31]

To avoid unintended hairpin formation within compute

computations, and outputs are linearly separable with a per-program-threshold, Supplementary Note S7.2.6.

It is tempting to interpret fast kinetics as evidence that scale-up to $N > 4$ is possible. As Supplementary Note S3.3 shows, if the SDC actually obeys the bind/replace kinetics proposed in Fig. 1, computations would take time merely bounded by $O(N^2)$; the time for all compute domain mismatches to disappear by random-walk to position N. However, theory and naive optimism need tempering by some practical challenges.

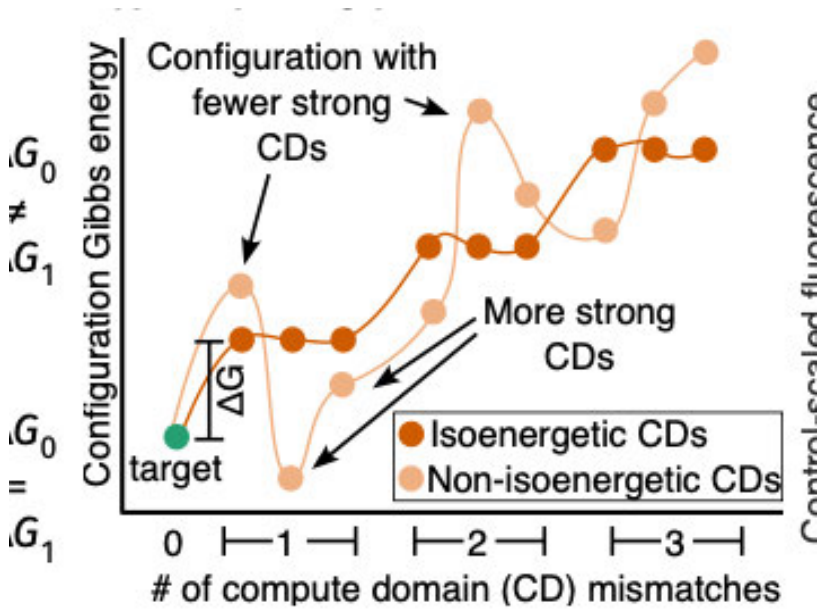

Another central ingredient here seems to be that the multiple "replace" pathways allow the system to effectively perform thermodynamic computation (as in reach the lowest free energy state/state with the best possible enthalpy). Again, this is a very interesting point but I am not completely sure how general this can be.

Reply. Yes, bind/replace kinetics is fully general, in the sense it is a sufficient form of kinetics to implement any finite state machine.

Action: To address this point we added text on kinetics to several parts of the main paper. The more technically correct real answer, but a level of detail unsuited to the main text perhaps, to the reviewers' question is to be found in the newly written SI Section S3.3. This gives an even stronger result than asked for: it shows that the SDC bind/replace kinetics gives the correct output to the computation and does so in no more than quadratic time of scaffold length. The time is a (loose) upperbound. We also simulated the process on four different simulators, the results of two of which are shown in Fig. S1 in SI Section S3.3. (Screenshots included to give an idea of what to look for.) However, did in fact include high level sentences in the main text that capture the spirt of the proof:

temperature-dependent, pathways.

Under bind/replace kinetics the expected computation time is upper-bounded by $O(N^2)$, the time for the leftmost compute domain mismatch (error) to disappear by a random walk to scaffold position N assuming a single anchor tile (Supplementary Note S3.3). However, although we defined one reasonable kinetic model, we intentionally do not experimentally enforce any particular kinetics, a *laissez-faire* approach that stands in contrast to DNA computing using toehold-mediated strand dis-

Also:

It is tempting to interpret fast kinetics as evidence that scale-up to $N > 4$ is possible. As Supplementary Note S3.3 shows, if the SDC actually obeys the bind/replace kinetics proposed in Fig. 1, computations would take time merely bounded by $O(N^2)$; the time for all compute domain mismatches to disappear by random-walk to position N. However, theory and naive optimism need tempering by some practical challenges.

And:

Fig. 6a, MULTIPLYBY3 and PARITY.

Intuition suggests equilibrium computation could be imprecise, since any desired output configuration competes with exponentially many off-target structures. We outline thermodynamic and kinetic arguments against this intuition for the SDC. Our thermodynamic design principles engineer the predicted target (MFE) structure to have probability approaching 1.0, driving the sum of probabilities of all other structures to 0 (Fig. 2f, Supplementary Note S4.3.1). Indeed, the correct SDC output was reported in all experiments. Notably, average completion/yield on typical-anneals, at scaffold length ≤ 4, was 95.3 % (SD=0.035) of strict controls with fewer strands and simpler equilibria. Mean yield at scaffold positions B and C was 98.1 % (SD=0.014), dropping to 94.4 % (SD=0.034) at D. Increasing competitive complexity from 2 to 8 did not significantly degrade yield for typical 3-hour anneals.

For larger N, mere thermodynamic favourability may not be sufficient: we need a nicely-shaped and traversable energy landscape. The abstract SDC tile model gives a stepped landscape, efficiently explorable by random walks that ratchet forward via compute domain mismatch repair. Hence, in theory and practice, we took a somewhat liberal approach to kinetics. The bind/replace model in Fig. 2b and Supplementary Note S3.3 begins a computation by guessing one of a large set of $2^{O(N)}$ initial states, yet by mere random walk completes in only $O(N^2)$ time. However, experimentally, we did not enforce this particular kinetics, nor any other: we simply annealed the system, hypothesising that something akin to bind/replace is happening at higher temperatures. We used a strand design without intentional kinetic traps (Fig. 2), but did not carry out design optimisations, nor did we seek to characterise and prevent off-pathway interactions, approaches common in typ-

In Section S3.3 , the reviewer can find mathematical and simulation details showing the bind/replace kinetics are sufficient to run SDC computations correctly:

S3.3 Bind/replace kinetics for the SDC tile model

In this section, we propose a putative kinetic model, building on the kinetics in Fig. 2b of the main text. Our goal is not to quantitatively explain the data, a task far beyond the scope of this paper, but rather to give a simple model that matches our intuition for how a perfectly-designed SDC might work and qualitatively shows agreement with our experiments. We leave it as future work to precisely parameterise this model or find a better one.[6]

Consider the tile-based model described in Fig. 2a–c of the main text, and more precisely defined in Definitions S3.1 and S3.2. The initial state consists of a free scaffold, with tiles in solution (in excess), and where bind and replace operations happen. Lemma S3.1 says the system eventually reaches a target state, next we consider the time to get there. We augment Definition S3.2 with rates:

Definition S3.3 (Bind/replace tile kinetics)**.** To Definition S3.2 we add rates for each step: The time for a bind or replace step is an exponential random variable with mean 1.

The resulting kinetic model is a continuous time Markov chain (CTMC), with the rate of the next event being the sum of the rates of all applicable events at that time instant.

Lemma S3.1 showed that any deterministic system (defined in Section S3.1) eventually goes to its target structure, the following lemma shows the expected time for that to happen under bind/replace kinetics. The energetic landscape is illustrated in Figure S1(a), and the artifically slow kinetic model used in the proof is simulated in Figure S1(b).

Lemma S3.2 (Completion time)**.** *For a deterministic SDC that obeys the hypothesis of Lemma S3.1, with 1 anchor tile that binds to the scaffold position 1, and $O(1)$ tiles for each other scaffold position, the expected time to reach the target is $O(N^2)$.*

[6] In fact we believe that at different temperatures, different forms of kinetics are possible, we'd conjecture for one important fast kinetics comes from a model like what is suggested here, and perhaps some of the slowest, and most irrelevant, coming from low-temperature toehold mediated strand displacement (as discussed in Section S4.3.2, with data in Figure S5).

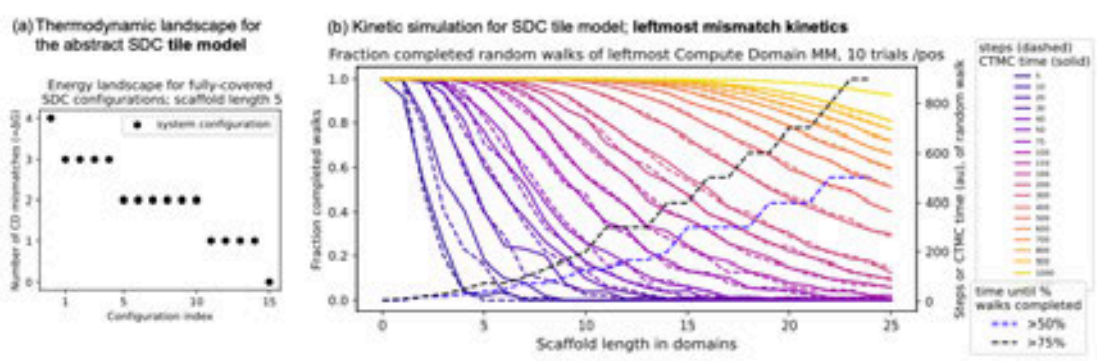

Figure S1: Two plots that illustrate the thermodynamics and kinetics of idealised SDC tile programs, such as BitCopy, assuming all domains are isoenergetic. These illustrate the proof of Lemma S3.2. (a) Simplified energy landscape for SDC tile model, assuming a tile on each scaffold position. Each additional mismatch gives an enthalpic penalty, assuming compute domains are strong enough and perfectly isoenergetic (so several other enthalpic and entropic complications can be ignored). (b) Kinetic simulations for a model that is at least as slow as, and likely slower than, the bind/replace kinetic model. Brightly coloured curves shows the fraction of completed random walks, of a mismatch starting at the leftmost position 0, on a length N scaffold, within a certain number of steps, or time. The random walk models the migration of the leftmost compute domain mismatch, assuming that it never meets another mismatch. This simplified model ignores speedups such as parallel cancelling of compute domain mismatches, hence this is a kind of worst-case kinetics for the SDC bind/replace, nevertheless finishes in expected time quadratic in scaffold length N. The black and blue dashed curves show, for a given scaffold position, how much time to wait until that % of walks have completed.

Proof. We prove the $O(N^2)$ time bound for a simpler artificially slow process, one that is necessarily at likely slower than the modelled process (but certainly no faster). First, we allow only bind steps, by (artificially) preventing any replace steps, until the scaffold is filled with tiles. For a scaffold of length $N \in \mathbb{N}$, the expected time to cover the scaffold with tiles is $O(\log N)$, since each tile binding event happens independently and in parallel. Only after covering the scaffold do we allow replacements to occur.

In the exponentially (in N) unlikely event we get a scaffolded structure with N tiles with all adjacent compute domains matching, we are done, but since that is unlikely we ignore that case and consider instead that there is at least one compute domain mismatch. Consider the leftmost such compute domain mismatch. We claim that the leftmost mismatch, over time, undergoes a random walk, that completes in $O(N^2)$ expected time, the proof is straightforward:

1. First, a compute domain mismatch between two tiles at positions 1 and 2 can not move to the left since there is only 1 anchor tile, which thus can not be removed by a replace step, hence we say that position 1 is a reflecting barrier.
2. Second, if the leftmost mismatch is between the tiles at positions $N-1$ and N, then there is always a replace step that replaces the tile at N with a matching tile which removes that mismatch completely; in other words position N is an absorbing position.
3. Third, if the leftmost mismatch is at some other position $1 < i < N$, it is repairable by a tile being replaced on the left or on the right of the mismatch: (1) if the replacement is to the left (respectively, right) and has a mismatch on its left at position $i-1$ (respectively, right at position $i+1$), we say the leftmost mismatch has taken a step to the left or, respectively right; else if (2) the new replacement tile (either at $i-1$ or $i+1$) has no mismatches on either of its compute domains then the leftmost mismatch of the system has either jumped to a new position $j > i$ (i.e. what was the second to left mismatch becomes the new leftmost mismatch), or we had started out with one mismatch and now there is no mismatch at all. Artificially, assuming the worst case where there are no jumps, the leftmost mismatch undergoes equiprobable step to the left or right.

Hence, in our artificially slowed-down model, the leftmost mismatch undergoes a random walk until absorbed at position N, which is well-known to take $O(N^2)$ expected time. Hence the expected completion time of the full system, without artificial suppression of steps, is also $O(N^2)$. □

15

Is annealing with some rate a pre-requisite ?

Reply: Yes. Annealing is certainly one of the best ways to find the ground (output) state and our we need slower annealing times as we scale our system up. We see reasonably good results within 14 hours for 25-bit ADDITION and our length 25 Isoenergetic-Bit-Copy system. Note that previous systems needed temperature holds, which is strictly harder for an experimentalist than annealing. The need for temperature holds is largely due to the fact these systems are out of equilibrium, if you anneal them to equilibrium you get a pile of junk (the components of the

systems bind in unintended ways). In fact, it has been a huge experimental convenience for us to be able to anneal our system.

As noted in the previous answer above, we gave arguments why completion time is O(*N*^2) (i.e. upper-bound) for a scaffold with *N* domains. That model is a simple random talk model that is necessarily slower than our proposed “bind/replace” kinetics in the paper.

We should add that for the experimental system, although the experiments show reasonably fast kinetics, it will take years of careful study to properly understand the kinetics our system–for context it has taken decades to learn about the kinetics of folding DNA origami.

Action:
Our scaled up experiments show that yes, annealing at slower rates helps systems of larger size (larger number of scaffold positions), this can be seen in the data in the new Figure 6, where 14 hr anneals give significantly better (closer to 0 or 1) results than 3 hr:

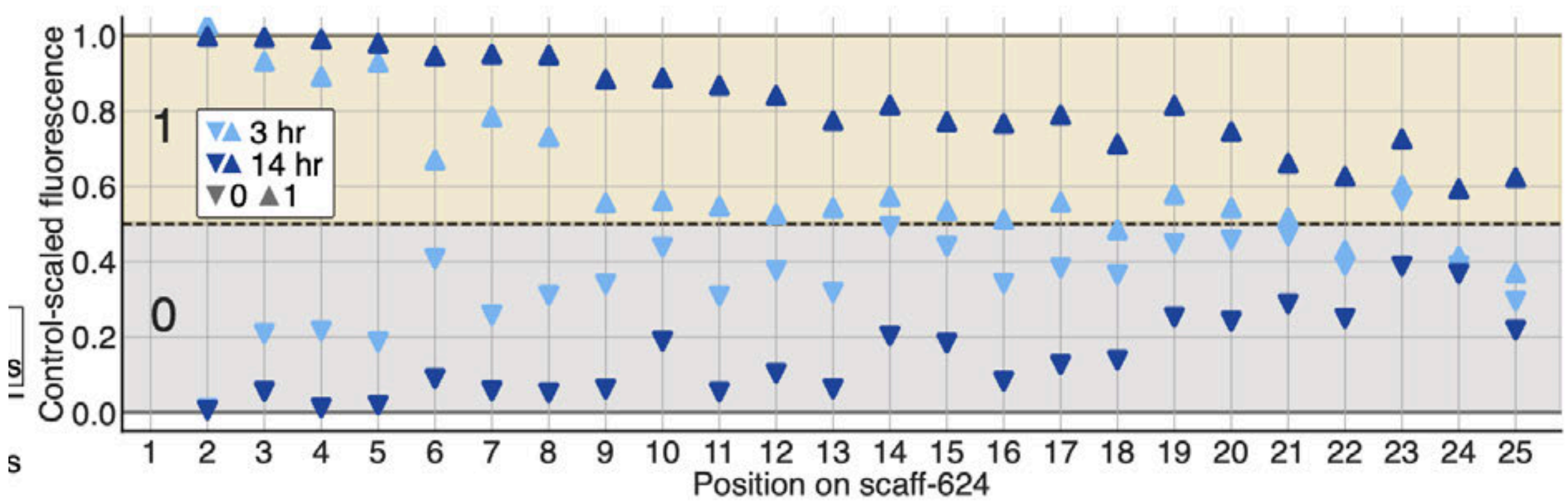

For context, typical DNA computation can take over a day, for smaller/easier computations (e.g. Woods, Doty et al Nature, 2019).   Some data from the SI (plotted slightly differently):

(c) Completion levels of anneals ISOENERGETIC BITCOPY: 3-hour, 8-hour and 14-hour

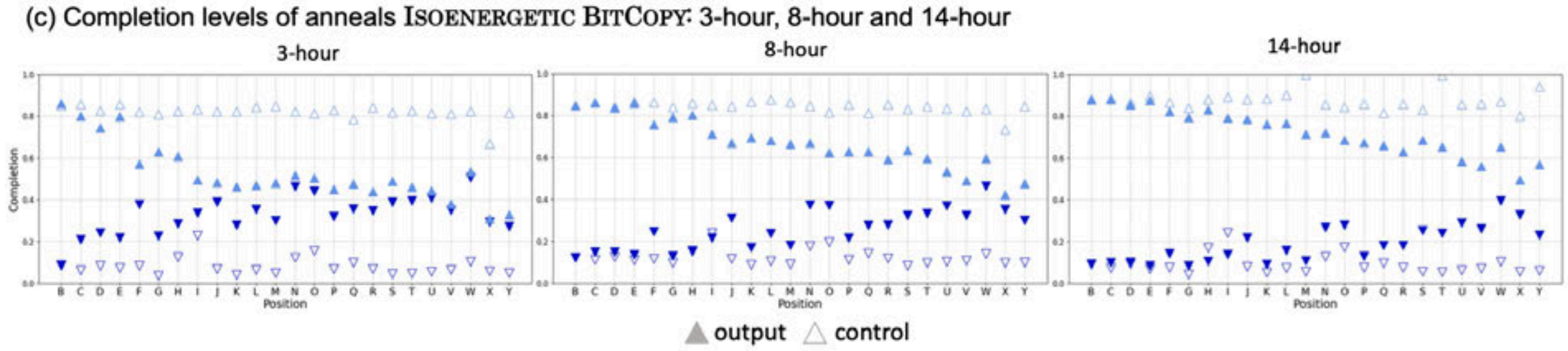

Other computations go much faster, this result shows ADDITION of two 25-bit numbers in an hour:

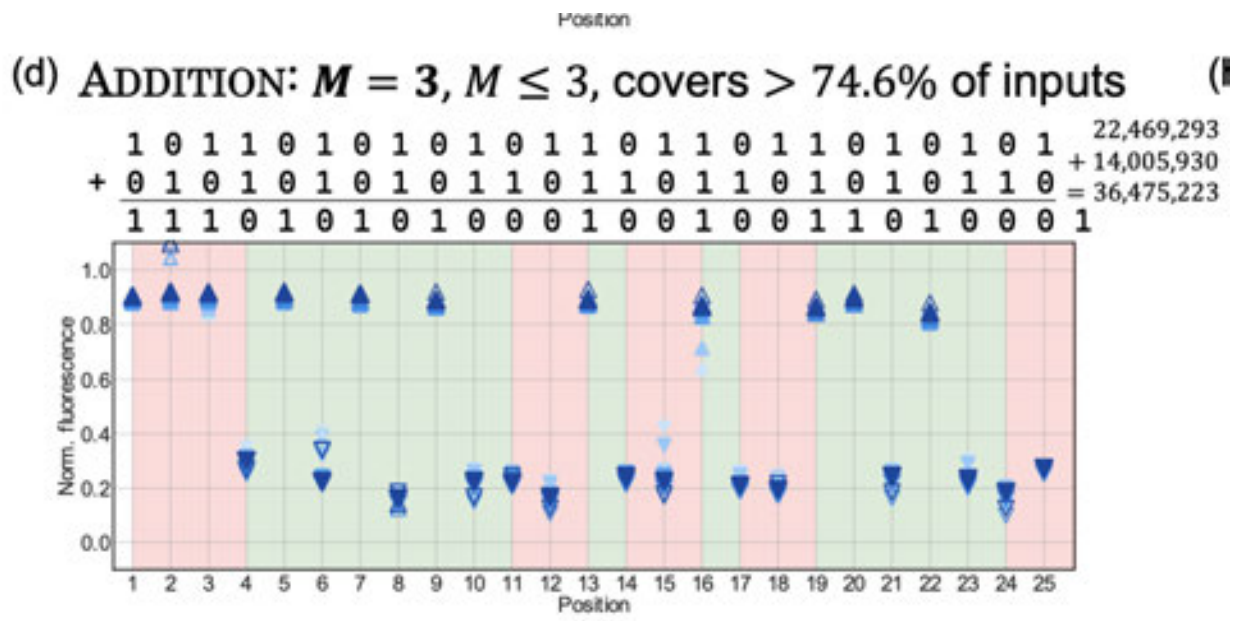

But depending on the “carry” structure of the inputs (or value of M), it can take longer. See Figure 6 in main text, or Figure S45 in the SI.

In asking these questions, I am partially motivated by a similar class of questions that emerge when dealing with self-assembly. There, kinetic traps are known to arrest formation of complex structures. It is known that in some cases a quench in protocols/temperature might help nucleate the correct structure. Is that similar to what the authors are proposing ? Explaining this will help (in my opinion) imrprove the readability of the paper and help it reach a broader range of readers.

Reply: Interesting, we also know of some systems that exploit temperature quenching. However, no, a temperature quench would not work with SDC; if the quench went to an extremely low temperature it would kinetically trap unintended logical errors (mismatching tiles). The example in Fig 2g, will take a few minutes to get through but we hope the payoff in understanding would be worth it, the idea is:

- Tiles bind randomly to the scaffold
- Tiles that mismatch their neighbours fall off, and are replaced by matching tiles
- Eventually the system settles down and there are no mismatches; that error-free structure result is the correct output for the computation

We have built multiple simulators of the process. Fig 2f shows the equilibrium of those structures, as calculated by the model in a related theory paper.

Action. We have put more emphasis on reducing technical terminology, and on explaining at a basic level some of one conjectured kinetics the SDC is undergoing. The new text, already mentioned above, takes a more mechanistic approach (by explaining about the energy landscape and random walks), as does the new Figure 6, that we hope will aid the reader.

I am happy to re-evaluate this work after the authors answer the above questions.

Reply: Thank you for the interesting questions. We welcome further feedback on improving the paper. The scale-up work brings many new aspects to it in particular, which should make the kinetics of our thermodynamic-favoured molecular computer more apparent in the data, and has motivated the new theory.

We would first like to thank the referees for the time and attention that they put in reviewing our manuscript. We are grateful for the feedback we received and for these enthusiastic assessments of our work.

Summary of reply to reviewers:

- We added scaffold sizes 50 and 100 in Fig 2F
- We completed the correction of our scaled-up ADDITION experiment

-------- Referees' comments ------
----- Referee #4 (Remarks to the Author):

I think this is exciting work, in particular because of the robustness and simplicity of the system compared to existing DNA computation approaches. Having read through the prior reviewers' comments and author responses, I have no major additional points. I have mainly suggestions/questions that may help improve readability/comprehensibility for a more general scientific reader.

Reply: We thank the referee for their kind words!

Fig 1F. Can the authors scale this theoretical analysis to larger numbers of scaffold positions to gain an understanding if there a practical limit arising from not being able to use a low enough equilibrium temperature (i.e. freezing conditions or kinetics too slow).

Reply: We included scaffold sizes 50 and 100 in Fig 2F, with melting temperatures still well above 60°C. We note that more detailed modelling/analysis would be required in the case of very long scaffolds, on the positive side, for example, SI Theorem S3.3 clearly suggests scaling compute domain length logarithmically with scaffold size, on the negative side it will be interesting to find out new modeling limitations as we scale further.

Related, comparing the error rates of Figure 6 c and d, it seems that using different compute domains across the tiles is important, as perhaps using identical compute domains for tiles at multiple positions as in BITcopy might be causing problems. In the discussion about scaling, it could be helpful to include theoretical discussion of how the diversity of compute domain sequence space may or may not impact scaling.

Reply: We have updated the discussion on this topic, including citing our new theory paper (Petrack, Evans et al, DNA32, 2026) which mathematically proves that the idea underlying IsoenergeticBitCopy can be applied to any SDC program. This means that the problem the reviewer is describing, i.e. use of repeated non-isoenergetic compute domains, is no longer a problem (at least theoretically).

Some typos (did not catch all, but noticed these): line 126: "level". 213: "distinct". 304: "in". Figure 6 caption: "prevents truncates".

Reply: Many thanks, we corrected them.

Fig 3e. Could the authors include a sentence or two in the methods or supplements, (I might have missed it), for why the fluorescence starts off above the normalized value of 1? Is there some basal level of quenching that occurs after an hour?

Reply: The reason for why fluorescence starts off above the normalized value of 1 is because in our data treatment (SI S7.2.3 and Figure S13) we apply a linear scaling so that the annealing curves end up (rather than start at) at the normalized fluorescence value of 1 when the expected algorithmic output is 1. Thank you for this comment; we better highlighted the sentence "*Normalised averaged repeats obtained after Step 2 are rescaled linearly so that y-axis value 0.832 becomes 1.0 and 0.033 becomes 0.0*" in SI S7.2.3.

I preface this question by saying I think I am missing something conceptually about all dna-based computation, and likely is my understanding issue. But potentially other readers coming from a more general background might have a similar question: Is the fact that the physical instantiation of the computer being the same as the input strands themselves (or at least including the input strands themselves) placing DNA computation in a different category than an FSM? Taking the ADDITION example, one would select a specific set of DNA oligos corresponding to that addition. This to me seems like you are using hardware/perhaps robotics to execute an initial operation that uses an algorithm to find those oligos and mix them. Then the oligos carry out a computation that essentially handles carryovers. Where in this whole process is the computation actually occurring? Is part of it actually being carried out in advance in the selection and physical selection and mixing of oligos? And how does this compare to the execution of computation using electronic hardware which seems more autonomous and general and not something that needs to be physically morphed for each different computation. I think I understand that this is a physical implementation of a FSM, but maybe the point I'm tripped up over is that for this implementation, the FSM is changing each time: in particular, most oligos from the library are being left out.

Reply: Thank you very much for this question! This was addressed deep in the SI of the previous version of this paper, and is a subtle point that we found difficult to get into in the (more general) main text; now the main text has a clearer pointer: "Supplementary Note S4 expands on SDC computational theory and explains why mixing input and program bits on the same tile is a computationally legitimate encoding."

In Section S4.4 "*Our tile set encoding does not cheat*" of the SI, "cheating" is defined as the reviewer describes it, and we show such cheating is not happening. In short, yes, a compilation step (choosing and mixing the oligos) happens in advance, but it is far too simple to be secretly doing the computation: it just translates the program and input into tiles, without ever "knowing" the answer (in the case of ADDITION, both possible carries 0 and 1 are selected for each position). The real work happens in the tube during annealing, where the strands compete and, in the case of ADDITION, actually determine the actual sequence of carry propagation (by selecting only one carry per position, the correct one). And this isn't just intuition: in S4.4 we formalise it and prove theoretically that the tile encoding step is provably too weak (FAC^0) to solve interesting problems on its own (like PARITY, Fig 4a). A simpler mathematical argument can also work for many problems also (re-run a program, but switch one tile for another, all other tiles along the scaffold need to correctly report the new correct answer which is potentially influence by the new tile).

I have a suggestion on providing more explanation in the presentation for a broader scientific audience. I personally would call myself peripherally aware of work in the DNA computing field, well informed about molecular biology, and an engineer by training, so I think I'm somewhat able to follow basic mathematical/physicist/computer scientist principles. Given that background, which I suspect would be representative of many who would be interested in this work but not steeped in computer science, I had substantial difficulty following what the "000, 000 with a bar" notations meant (I realize it is explained in the supplement but would be quite buried for a general reader), what the "program instructions" versus "input bits" meant, and I did not understand the notation of the finite state machines (especially figures 4c and 4d). I know it is always difficult to cater to broad audiences, but I think a sentence here or there to help would help, despite the word limits.

Reply: Thank you very much for this suggestion: we moved this technical point to the Methods, which allowed expanding the text to explicitly explain that 000 and 000-hat are domains with non-complementary sequences, which in turn avoids hairpin formation. As noted, more details are also in the SI.

Referee #4 (Remarks on code availability):

The code base is well organized and I was able to run the code, although I did not extensively test it for errors.

Reply: Thank you!

----- Referee #5 (Remarks to the Author):

1. Summary of the key results:

The manuscript presents a DNA-based implementation of thermodynamically favored computation. Rather than driving computation through a fixed sequence of fuel-consuming kinetic steps, the authors design molecular systems in which the desired computational output is favored at equilibrium. The authors demonstrate that their scaffolded DNA computer (SDC) systems can compute accurately and rapidly across multiple programs. In the revised manuscript they have substantially scaled up the system, included new theory, new simulations, and a much clearer discussion of energy landscapes, kinetic traps, and the role of bind/replace pathways.

2. Originality and significance:

The theoretic idea of thermodynamic computing is not new, and the manuscript acknowledges this clearly. That said, I find the specific experimental realization presented here to be highly original. The novelty is not simply that computation can be mapped to a thermodynamic minimum, but that the authors have built a fast, reliable molecular implementation that scales substantially beyond a small proof of concept.

Having access to the first round of reviews, I can see that the authors have clearly gone to great lengths to improve the manuscript based on the reviewer feedback they received. They have directly addressed the questions about scalability, and they have modified the framing of their work to improve the contextualization. I find it quite compelling that the authors rose to meet the scalability challenge presented to them and implemented several 25-position (arguably more than 25 bit) algorithms! As a comparison, Woods' wonderful prior work published in Nature implemented 6-bit algorithms. Not only does the current manuscript represent a substantial increase in computational scale, but the drastic increase in speed also further strengthens the significance of the result.

The revised manuscript is also much better about not overclaiming. I do not read the current version as claiming that thermodynamic computation will replace silicon computers, quantum computers, or driven molecular computation more generally. Rather, the paper now makes a more defensible and more interesting claim: that molecular energy landscapes can be programmed and navigated to perform computation in a way that is experimentally practical and scalable beyond toy examples.

That conclusion is well supported, and in this revised form I think the work is appropriate for Nature.

3. Conclusions: robustness, validity, reliability

I have read most of the SI and I can't praise it enough. It is a fantastic example of "full-stack" nanotechnology development. Multiple levels of abstraction supporting a concept with beautiful theoretical work and leading it all the way to implementation.

I also find the comparison to DNA origami to be quite appropriate. Their approach has a similar practical appeal: simple strand-level design rules, good performance on useful timescales for reasonable systems, and relatively economical implementation (including the ability to use many unpurified strands without sacrificing yield). Of course, fully customized long synthetic scaffolds may become expensive for larger implementations, but the overall architecture feels experimentally accessible in much the same way that DNA origami was largely adopted because it "just works".

The authors are very clear that they don't enforce (or even rely) on particular kinetics. They hypothesize that many different kinetic paths leading to the same equilibrium state are what allow the system to compute so accurately and quickly. However, they are very clear that they mean to invoke discussion on this, and don't claim for it to be generally true (it just seems to work for their SDC schema).

Reply: We are sincerely grateful for the referee's generous words of encouragement.

4. Suggested improvements:

The most important revision I recommend is to complete the correction to the ADDITION experiments. Because the ADDITION experiments are among the central scale-up

demonstrations in the revised manuscript, the authors should complete the corrected experiments and update the appropriate sections of the paper.

Reply: Context: The previous ADDITION experiments were missing one data point out of 25, the new repeated experiments have that data point. We absolutely agree with this comment and have updated accordingly scaled-up ADDITION data in Fig 6a.

I concur with a previous reviewer that it is a shame that the very nice thermodynamic computing theory in the SI does not appear in the main text. However, I also think that the current version is well-suited for the general audience of Nature and the substantial rewrite required to incorporate those SI sections into the paper is likely not worth it.

One conceptual clarification that would be helpful is whether different output methods affect composability (whatever that may mean in a thermodynamic computing setting). For example, does using an output bit versus an output string change how readily the output of one computation can be used as the input to a subsequent computation? This does not affect my assessment of the current results, but it is related to scalability in some sense, and it may help readers understand how the architecture might be incorporated into larger computational workflows.

Reply: Composability in thermodynamically favoured molecular computers is a fascinating question! It currently seems clear that on the same scaffold, SDC computations are inherently composable (e.g. if scaffold domains A,B,C are set to run ADDITION and domains D,E,F to run PARITY); however, composing SDC computations that run on separate scaffolds is open and constitutes a very interesting area of research for future work.

Minor errata:

Line 271: “We designed a …” appears to refer to multiple scale-up tests. Consider changing “a” to “some,” or otherwise revising the sentence for number agreement.

Line 304: “An M = 3 computation in is …” Remove “in.”

Lines 306–307: “covers over >97%” is redundant. Use either “covers over 97%” or “covers >97%.”

Figure 6 caption: “which prevents truncates error pathways” is unclear. Perhaps the intended phrase was “prevents/truncates error pathways,” or perhaps only one of those verbs was intended.

Acknowledgements: “We thank Ahmed Shalaby his fast …” appears to be missing “for.”

Reply: Many thanks we fixed these issues!

5. References: appropriate credit to previous work?

Overall, the background literature review is quite thorough, both in the main text and in the SI, and tells a lovely story of how this work fits into a broader effort to program molecular energy landscapes to compute. In particular, I appreciated the effort the authors put into S2 to connect the work to several neighboring literatures without making the background feel like a list of citations; instead, the discussion helps clarify what is genuinely new here and what longstanding ideas the manuscript builds on.

Reply: Many thanks! We are happy to hear the referee appreciated this. It can be difficult to set the context properly in a short space in the main text.

I do have one citation recommendation: Very recent work [1] seems to employ the thermal-cycling-reset concept together with the strand-displacement input-replacement technique to run new computations in a way that resembles the authors' renewable programs. However, [1]'s rechargeable circuits keep resetting back into a kinetic trap, rather than stable equilibrium. So, while the mechanisms and framing are distinct, [1] seems like a conceptual cousin of this work and would likely be a relevant addition to S8.1.

[1] Song, Tianqi and Qian, Lulu. "Heat-rechargeable computation in DNA logic circuits and neural networks." Nature 646.8084 (2025): 315-322.

Reply: Thank you very much for this very relevant suggestion; this paper came out after we made our own work public on bioarxiv and after we submitted to this journal. We now cite it in the main text, and Integrated it into the discussion of relevant literature on renewable DNA computing.

Many thanks to both referees!

We would first like to thank the referees for the time and attention that they put in reviewing our manuscript. We are grateful for the feedback we received and for these enthusiastic assessments of our work.

Summary of reply to reviewers:

- We added scaffold sizes 50 and 100 in Fig 2F
- We completed the correction of our scaled-up ADDITION experiment

-------- Referees' comments ------
----- Referee #4 (Remarks to the Author):

I think this is exciting work, in particular because of the robustness and simplicity of the system compared to existing DNA computation approaches. Having read through the prior reviewers' comments and author responses, I have no major additional points. I have mainly suggestions/questions that may help improve readability/comprehensibility for a more general scientific reader.

Reply: We thank the referee for their kind words!

Fig 1F. Can the authors scale this theoretical analysis to larger numbers of scaffold positions to gain an understanding if there a practical limit arising from not being able to use a low enough equilibrium temperature (i.e. freezing conditions or kinetics too slow).

Reply: We included scaffold sizes 50 and 100 in Fig 2F, with melting temperatures still well above 60°C. We note that more detailed modelling/analysis would be required in the case of very long scaffolds, on the positive side, for example, SI Theorem S3.3 clearly suggests scaling compute domain length logarithmically with scaffold size, on the negative side it will be interesting to find out new modeling limitations as we scale further.

Related, comparing the error rates of Figure 6 c and d, it seems that using different compute domains across the tiles is important, as perhaps using identical compute domains for tiles at multiple positions as in BITcopy might be causing problems. In the discussion about scaling, it could be helpful to include theoretical discussion of how the diversity of compute domain sequence space may or may not impact scaling.

Reply: We have updated the discussion on this topic, including citing our new theory paper (Petrack, Evans et al, DNA32, 2026) which mathematically proves that the idea underlying IsoenergeticBitCopy can be applied to any SDC program. This means that the problem the reviewer is describing, i.e. use of repeated non-isoenergetic compute domains, is no longer a problem (at least theoretically).

Some typos (did not catch all, but noticed these): line 126: "level". 213: "distinct". 304: "in". Figure 6 caption: "prevents truncates".

Reply: Many thanks, we corrected them.

Fig 3e. Could the authors include a sentence or two in the methods or supplements, (I might have missed it), for why the fluorescence starts off above the normalized value of 1? Is there some basal level of quenching that occurs after an hour?

Reply: The reason for why fluorescence starts off above the normalized value of 1 is because in our data treatment (SI S7.2.3 and Figure S13) we apply a linear scaling so that the annealing curves end up (rather than start at) at the normalized fluorescence value of 1 when the expected algorithmic output is 1. Thank you for this comment; we better highlighted the sentence "*Normalised averaged repeats obtained after Step 2 are rescaled linearly so that y-axis value 0.832 becomes 1.0 and 0.033 becomes 0.0*" in SI S7.2.3.

I preface this question by saying I think I am missing something conceptually about all dna-based computation, and likely is my understanding issue. But potentially other readers coming from a more general background might have a similar question: Is the fact that the physical instantiation of the computer being the same as the input strands themselves (or at least including the input strands themselves) placing DNA computation in a different category than an FSM? Taking the ADDITION example, one would select a specific set of DNA oligos corresponding to that addition. This to me seems like you are using hardware/perhaps robotics to execute an initial operation that uses an algorithm to find those oligos and mix them. Then the oligos carry out a computation that essentially handles carryovers. Where in this whole process is the computation actually occurring? Is part of it actually being carried out in advance in the selection and physical selection and mixing of oligos? And how does this compare to the execution of computation using electronic hardware which seems more autonomous and general and not something that needs to be physically morphed for each different computation. I think I understand that this is a physical implementation of a FSM, but maybe the point I'm tripped up over is that for this implementation, the FSM is changing each time: in particular, most oligos from the library are being left out.

Reply: Thank you very much for this question! This was addressed deep in the SI of the previous version of this paper, and is a subtle point that we found difficult to get into in the (more general) main text; now the main text has a clearer pointer: "Supplementary Note S4 expands on SDC computational theory and explains why mixing input and program bits on the same tile is a computationally legitimate encoding."

In Section S4.4 "*Our tile set encoding does not cheat*" of the SI, "cheating" is defined as the reviewer describes it, and we show such cheating is not happening. In short, yes, a compilation step (choosing and mixing the oligos) happens in advance, but it is far too simple to be secretly doing the computation: it just translates the program and input into tiles, without ever "knowing" the answer (in the case of ADDITION, both possible carries 0 and 1 are selected for each position). The real work happens in the tube during annealing, where the strands compete and, in the case of ADDITION, actually determine the actual sequence of carry propagation (by selecting only one carry per position, the correct one). And this isn't just intuition: in S4.4 we formalise it and prove theoretically that the tile encoding step is provably too weak (FAC^0) to solve interesting problems on its own (like PARITY, Fig 4a). A simpler mathematical argument can also work for many problems also (re-run a program, but switch one tile for another, all other tiles along the scaffold need to correctly report the new correct answer which is potentially influence by the new tile).

I have a suggestion on providing more explanation in the presentation for a broader scientific audience. I personally would call myself peripherally aware of work in the DNA computing field, well informed about molecular biology, and an engineer by training, so I think I'm somewhat able to follow basic mathematical/physicist/computer scientist principles. Given that background, which I suspect would be representative of many who would be interested in this work but not steeped in computer science, I had substantial difficulty following what the "000, 000 with a bar" notations meant (I realize it is explained in the supplement but would be quite buried for a general reader), what the "program instructions" versus "input bits" meant, and I did not understand the notation of the finite state machines (especially figures 4c and 4d). I know it is always difficult to cater to broad audiences, but I think a sentence here or there to help would help, despite the word limits.

Reply: Thank you very much for this suggestion: we moved this technical point to the Methods, which allowed expanding the text to explicitly explain that 000 and 000-hat are domains with non-complementary sequences, which in turn avoids hairpin formation. As noted, more details are also in the SI.

Referee #4 (Remarks on code availability):

The code base is well organized and I was able to run the code, although I did not extensively test it for errors.

Reply: Thank you!

----- Referee #5 (Remarks to the Author):

1. Summary of the key results:

The manuscript presents a DNA-based implementation of thermodynamically favored computation. Rather than driving computation through a fixed sequence of fuel-consuming kinetic steps, the authors design molecular systems in which the desired computational output is favored at equilibrium. The authors demonstrate that their scaffolded DNA computer (SDC) systems can compute accurately and rapidly across multiple programs. In the revised manuscript they have substantially scaled up the system, included new theory, new simulations, and a much clearer discussion of energy landscapes, kinetic traps, and the role of bind/replace pathways.

2. Originality and significance:

The theoretic idea of thermodynamic computing is not new, and the manuscript acknowledges this clearly. That said, I find the specific experimental realization presented here to be highly original. The novelty is not simply that computation can be mapped to a thermodynamic minimum, but that the authors have built a fast, reliable molecular implementation that scales substantially beyond a small proof of concept.

Having access to the first round of reviews, I can see that the authors have clearly gone to great lengths to improve the manuscript based on the reviewer feedback they received. They have directly addressed the questions about scalability, and they have modified the framing of their work to improve the contextualization. I find it quite compelling that the authors rose to meet the scalability challenge presented to them and implemented several 25-position (arguably more than 25 bit) algorithms! As a comparison, Woods' wonderful prior work published in Nature implemented 6-bit algorithms. Not only does the current manuscript represent a substantial increase in computational scale, but the drastic increase in speed also further strengthens the significance of the result.

The revised manuscript is also much better about not overclaiming. I do not read the current version as claiming that thermodynamic computation will replace silicon computers, quantum computers, or driven molecular computation more generally. Rather, the paper now makes a more defensible and more interesting claim: that molecular energy landscapes can be programmed and navigated to perform computation in a way that is experimentally practical and scalable beyond toy examples.

That conclusion is well supported, and in this revised form I think the work is appropriate for Nature.

3. Conclusions: robustness, validity, reliability

I have read most of the SI and I can't praise it enough. It is a fantastic example of "full-stack" nanotechnology development. Multiple levels of abstraction supporting a concept with beautiful theoretical work and leading it all the way to implementation.

I also find the comparison to DNA origami to be quite appropriate. Their approach has a similar practical appeal: simple strand-level design rules, good performance on useful timescales for reasonable systems, and relatively economical implementation (including the ability to use many unpurified strands without sacrificing yield). Of course, fully customized long synthetic scaffolds may become expensive for larger implementations, but the overall architecture feels experimentally accessible in much the same way that DNA origami was largely adopted because it "just works".

The authors are very clear that they don't enforce (or even rely) on particular kinetics. They hypothesize that many different kinetic paths leading to the same equilibrium state are what allow the system to compute so accurately and quickly. However, they are very clear that they mean to invoke discussion on this, and don't claim for it to be generally true (it just seems to work for their SDC schema).

Reply: We are sincerely grateful for the referee's generous words of encouragement.

4. Suggested improvements:

The most important revision I recommend is to complete the correction to the ADDITION experiments. Because the ADDITION experiments are among the central scale-up

demonstrations in the revised manuscript, the authors should complete the corrected experiments and update the appropriate sections of the paper.

Reply: Context: The previous ADDITION experiments were missing one data point out of 25, the new repeated experiments have that data point. We absolutely agree with this comment and have updated accordingly scaled-up ADDITION data in Fig 6a.

I concur with a previous reviewer that it is a shame that the very nice thermodynamic computing theory in the SI does not appear in the main text. However, I also think that the current version is well-suited for the general audience of Nature and the substantial rewrite required to incorporate those SI sections into the paper is likely not worth it.

One conceptual clarification that would be helpful is whether different output methods affect composability (whatever that may mean in a thermodynamic computing setting). For example, does using an output bit versus an output string change how readily the output of one computation can be used as the input to a subsequent computation? This does not affect my assessment of the current results, but it is related to scalability in some sense, and it may help readers understand how the architecture might be incorporated into larger computational workflows.

Reply: Composability in thermodynamically favoured molecular computers is a fascinating question! It currently seems clear that on the same scaffold, SDC computations are inherently composable (e.g. if scaffold domains A,B,C are set to run ADDITION and domains D,E,F to run PARITY); however, composing SDC computations that run on separate scaffolds is open and constitutes a very interesting area of research for future work.

Minor errata:

Line 271: “We designed a …” appears to refer to multiple scale-up tests. Consider changing “a” to “some,” or otherwise revising the sentence for number agreement.

Line 304: “An M = 3 computation in is …” Remove “in.”

Lines 306–307: “covers over >97%” is redundant. Use either “covers over 97%” or “covers >97%.”

Figure 6 caption: “which prevents truncates error pathways” is unclear. Perhaps the intended phrase was “prevents/truncates error pathways,” or perhaps only one of those verbs was intended.

Acknowledgements: “We thank Ahmed Shalaby his fast …” appears to be missing “for.”

Reply: Many thanks we fixed these issues!

5. References: appropriate credit to previous work?

Overall, the background literature review is quite thorough, both in the main text and in the SI, and tells a lovely story of how this work fits into a broader effort to program molecular energy landscapes to compute. In particular, I appreciated the effort the authors put into S2 to connect the work to several neighboring literatures without making the background feel like a list of citations; instead, the discussion helps clarify what is genuinely new here and what longstanding ideas the manuscript builds on.

Reply: Many thanks! We are happy to hear the referee appreciated this. It can be difficult to set the context properly in a short space in the main text.

I do have one citation recommendation: Very recent work [1] seems to employ the thermal-cycling-reset concept together with the strand-displacement input-replacement technique to run new computations in a way that resembles the authors' renewable programs. However, [1]'s rechargeable circuits keep resetting back into a kinetic trap, rather than stable equilibrium. So, while the mechanisms and framing are distinct, [1] seems like a conceptual cousin of this work and would likely be a relevant addition to S8.1.

[1] Song, Tianqi and Qian, Lulu. "Heat-rechargeable computation in DNA logic circuits and neural networks." Nature 646.8084 (2025): 315-322.

Reply: Thank you very much for this very relevant suggestion; this paper came out after we made our own work public on bioarxiv and after we submitted to this journal. We now cite it in the main text, and Integrated it into the discussion of relevant literature on renewable DNA computing.

Many thanks to both referees!
